# PRER: A patient representation with pairwise relative expression of proteins on biological networks

**DOI:** 10.1371/journal.pcbi.1008998

**Published:** 2021-05-26

**Authors:** Halil İbrahim Kuru, Mustafa Buyukozkan, Oznur Tastan

**Affiliations:** 1 Department of Computer Engineering, Bilkent University, Ankara, Turkey; 2 Faculty of Natural Sciences and Engineering, Sabanci University, Istanbul, Turkey; University Medical Center Göttingen, GERMANY

## Abstract

Changes in protein and gene expression levels are often used as features in predictive modeling such as survival prediction. A common strategy to aggregate information contained in individual proteins is to integrate the expression levels with the biological networks. In this work, we propose a novel patient representation where we integrate proteins’ expression levels with the protein-protein interaction (PPI) networks: Patient representation with PRER (Pairwise Relative Expressions with Random walks). PRER captures the dysregulation patterns of proteins based on the neighborhood of a protein in the PPI network. Specifically, PRER computes a feature vector for a patient by comparing the source protein’s expression level with other proteins’ levels that are within its neighborhood. The neighborhood of the source protein is derived by biased random-walk strategy on the network. We test PRER’s performance in survival prediction task in 10 different cancers using random forest survival models. PRER yields a statistically significant predictive performance in 9 out of 10 cancers when compared to the same model trained with features based on individual protein expressions. Furthermore, we identified the pairs of proteins that their interactions are predictive of patient survival but their individual expression levels are not. The set of identified relations provides a valuable collection of protein biomarkers with high prognostic value. PRER can be used for other complex diseases and prediction tasks that use molecular expression profiles as input. PRER is freely available at: https://github.com/hikuru/PRER.

## Author summary

Cancer remains to be one of the most prevalent and challenging diseases to treat. Cancer is a complex disease with several disrupted molecular mechanisms at play. The protein expression level is a fundamental indicator of how the molecular mechanisms are altered in each tumor. Predicting patient survival based on the changes is essential for understanding the cancer mechanisms and arriving at patient-specific treatment plans. For this task, existing machine learning models are used, such as random survival forest, which requires a feature-based representation of each patient based on her tumors. Most of these models use the individual molecular quantities of the tumors. However, cancer is a complex disease in which molecular mechanisms are dysregulated in various ways. In this work, we present a new patient representation scheme in which we integrate each tumor’s protein expression levels with their neighboring proteins’ expression levels in a protein-protein interaction network to capture patient-specific dysregulation patterns. Our results suggest that proteins’ relative expressions are more predictive than their individual expressions. We also analyze which of the protein interactions are more predictive of patient survival. The identified set of important protein interactions can be potentially used for cancer prognosis.

This is a *PLOS Computational Biology* Methods paper.

## Introduction

With the advances in sequencing technologies, large-scale molecular profiling of patients has become possible. The comprehensive profiling of cancer patients, along with their clinical data, presents an opportunity to gain deeper insights into cancer and develop prediction tools for disease outcome. Machine learning has been an instrumental tool in various studies to realize this aim. In these studies, patients are often represented with their molecular profiles, such as protein or gene expressions. For example, Yuan et al. [1] assess the utility of different types of molecular data for survival prediction where miRNA, protein, or mRNA expressions were considered.. Similar approaches are followed by others for different clinical outcomes [2–4].

Genes and proteins interact to carry out their functional roles in the cell. Phenotypes arise from these functional interactions. Based on this basic principle, alternative approaches where the patient molecular data are integrated with cataloged molecular interactions, based on prior research, have been proposed (reviewed in [5] and [6]). Incorporating prior knowledge as the network of interactions helps to aggregate the information contained in each protein or gene in a biologically principled way. Integration of the expression levels of genes/proteins and their interactions are used in multiple studies [7–11]. Chuang et al. [8] are among the first to use this approach. They identify discriminant and highly altered subnetworks of interactions using gene expressions and use these subnetworks for metastasis prediction. By assessing the association of pathways and transcription factors with overall survival, Crijns et al. [10] identify signaling pathways and transcription factors that contribute to the outcome of ovarian cancer. Taylor et al. [9] integrate a PPI network with a co-expression network and report that the genes with dysregulated neighbors in the network are potential prognostic markers. NetBank [12] uses gene expressions and prior knowledge network to rank genes according to their relevance to the outcome of pancreatic cancer. In an alternative approach, Wang and Liu [13] use the topological importance of the proteins in the network to reweight them in random survival forest sampling. Some methods have used this idea for other types of omic profiles [6]. For example, Hofree et al. [7] integrates mutation data with PPI network for patient stratification through network propagation that diffuses gene-level mutations over the PPI network. These studies integrate molecular data with the network by summation or diffusion of the signal; however, do not consider relative expressions of proteins or genes with respect to each other.

Few studies use the pairwise comparisons of molecular measurements instead of the aggregation of expression levels. Geman et al. [14] proposes pairwise ranks of mRNA expression levels for tumor identification and prediction of treatment response. Magen et al. [15] use pairwise combinations of expression levels to predict survival and report related gene pairs. These methods, however, do not make use of the prior knowledge available in the biological networks.

In this work, we explore a method that combines the two aspects discussed above; network integration and pairwise comparison of expression levels. Pairwise Rank Expressions with Random walks (PRER) is a novel molecular representation method that considers the relative expression of a protein within its neighborhood on the PPI network. A given protein’s neighborhood is defined based on the biased random walk search on the PPI network. Pairwise relations within the known neighborhood of molecules offer a direct interpretation of molecular dysregulation patterns in the context of known protein interactions. Additionally, we present a method to analyze pairs that are predictive because of their pairwise comparisons.

We compute PRER representation using protein expression data obtained from patient tumors and used it for survival prediction in ten cancers cataloged in the Cancer Genome Atlas (TCGA) project [16]. When compared to the standard model that individual protein expressions are used, PRER yields a statistically significant improvement in 9 of the 10 cancer types. PRER is also shown to perform better against two network-based competitive methods. Additionally, PRER unveils predictive proteins and their interactions concerning the known PPIs. We also investigate proteins that are deemed significant solely based on their interactions.

## Methods

### PRER feature representation

PRER constructs a vector-based patient representation by integrating the patients’ molecular expression profiles and the PPI network. In this work, we use protein expression data to calculate PRER.

Let *G* = (*V*, *E*) be a given PPI network, where *V* is the set of vertices representing the proteins, and *E* is the set of edges that exist between proteins if they are known to interact. Let *U* ⊆ *V* be the proteins that are measured in the data set. The nodes with the protein expression data, *U*, represent the source proteins. Given *G*, *U*, and patient expression data over *U*, the output of PRER for a patient *k* is a feature vector, **x**^(*k*)^ ∈ *R*^*s*^, that contains the pairwise comparisons encoded as 1 and -1’s. Here, *s* denotes the size of the pairwise comparisons, which will be clarified in the following sections. Below we detail the steps of PRER.

#### Step 1. Obtaining a protein’s neighborhood on the protein interaction network

For each source protein in *U*, we first define a neighborhood, *N*_*u*_, which is the set of proteins proximal to the source protein *u* on *G*. To obtain the neighborhood of a node in the graph, a set of random walks is generated. For every source node *u* ∈ *U*, we sample neighbors of the source node with a strategy similar to the one in the node2vec [17] algorithm. A random walk with a fixed length of *l* starting at source node *u* is generated based on the following distribution:

$$\begin{array}{c} P\left(c_{i}=x|c_{i-1}=v\right)=\{\begin{array}{cc} \frac{\pi_{vx}}{Z} & \text{if}\;(v,x)\;\epsilon \;E\\ 0 & \text{otherwise}\; \end{array} \end{array}$$ (1)

Here, *c*_*i*_ denotes the *i*-th node in the walk and *c*_0_ = *u*. Z is the normalization constant. *P*(*c*_*i*_ = *x*∣*c*_*i*−1_ = *v*) is the transition probability on edge (*v*, *x*), where the current node is *v*, the next node to visit is *x*. The transition probability depends on the function *π*, and it is defined as:

$$\begin{array}{c} \pi_{vx}=\alpha_{pq}\left(t,x\right)*w_{vx}, \end{array}$$ (2)

where *w*_*vx*_ is the edge weight between nodes *v* and *x*; *t* is the previous node visited. In this work, we use an unweighted PPI network and, thus, we set *w*_*vx*_ = 1. *α*_*pq*_(*t*, *x*) is the random walk bias which is defined by Eq 3 based on the parameters *p* and *q* and the shortest path distance between nodes *t* and *x*, *d*_*tx*_:

$$\begin{array}{c} \alpha_{pq}\left(t,x\right)=\{\begin{array}{cc} \frac{1}{p} & \text{if}\;d_{tx}=0\\ 1 & \text{if}\;d_{tx}=1\\ \frac{1}{q} & \text{if}\;d_{tx}=2 \end{array} \end{array}$$ (3)

This bias controls the different search strategies to sample the next visited nodes. We use two different search methods: depth-first sampling (DFS) and breadth-first sampling (BFS), as in [17]. BFS samples the nodes from the nearby nodes, whereas DFS samples the nodes sequentially by gradually increasing the distance from a source node. Parameters *p* and *q* adjust random-walk strategy between BFS and DFS. With a high *q* value, sampled nodes in the random walk are aligned with BFS and get a local view over the source node. A small *q* value aligns random walk with DFS to explore a global view of the network. *p* controls the chance of revisiting the nodes. A high value of *p* decreases the probability of revisiting the already visited nodes, whereas a small value of *p* steers the random walk towards the source node.

**Fig 1:**
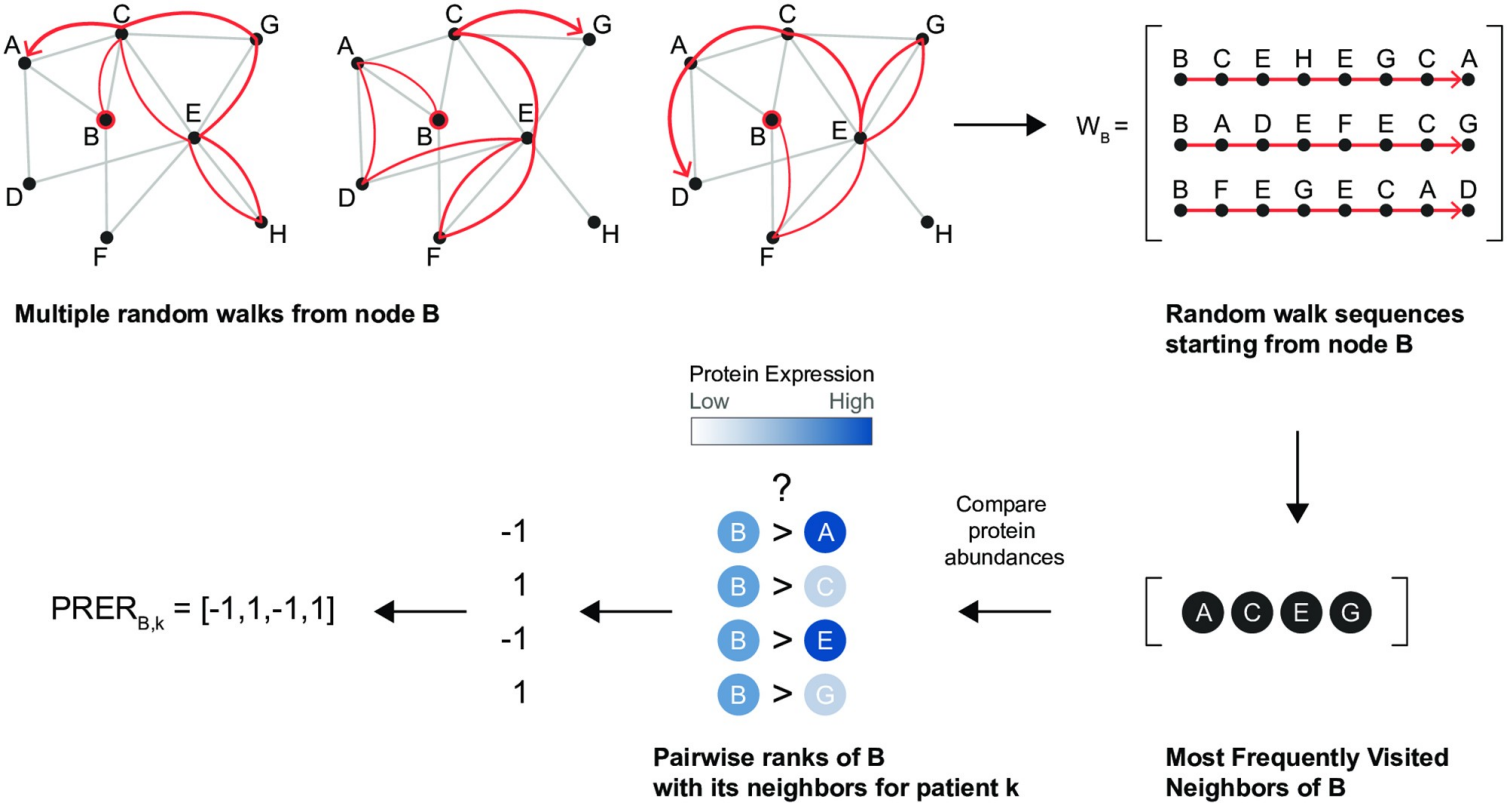
Illustration to show how the PRER representation is obtained for a single source node, node B. The nodes in the graph are proteins, edges exist if they interact in the PPI network. First, several random walks are generated that starts at node B as in [17]. These random walks are stored in *W*_*B*_ and used to define the neighborhood of *B*, *N*_*B*_. Only the most frequently visited nodes are included in the set of neighbors of B. Then, the pairwise comparison of the neighborhood proteins in terms of their protein expression quantities is used to form a representation of the patient for node B and its neighborhood. The figure shows the features generated for a single protein. This procedure is repeated for all source proteins, and the resulting vectors are concatenated.

This biased random walk strategy has two additional parameters: (i) walk length *l* and (ii) the number of random walks *r*. We select these parameters based on the parameter sensitivity analysis at node2vec [17]. The parameters *p* and *q* are used as *p* = 0.25, *q* = 0.25 in our random walk generation. *p* = 1, *q* = 1 leads uniform random walks are generated without any bias as stated in [17]. A small *q* value is used to bias the random walks to capture the network’s global view, while a small *p* value is used to capture the community around the source node *u*. With these given values, random walks are inclined to capture the communities inside the network. By using fixed-length (*l* = 100) random walks, we sample a neighborhood for a given source node, *u*. Multiple random walks per source node are generated so that different neighborhoods are sampled for each node. We sampled random walks 18 times, and these are stored in *W*_*B*_ (see Fig 1). The frequency of nodes in the multiple neighborhoods is calculated, and the nodes that are involved in more than one random walk are selected as the neighborhood genes. Later, we analyzed how the choice of these parameter assignments affects the results.

#### Step 2. Feature representation based on pairwise rank of neighborhood genes

At the end of step one, we arrive at the neighborhood of the protein *i*, which we denote as *N*_*i*_. Some neighbors lack measurements, and we define the subset of neighbor proteins with accompanying measurements as *M*_*i*_ ∈ *N*_*i*_ ∩ *U*. Next, for a protein *i*, we generate pairwise rank features with every protein *i* ∈ *M*_*i*_ as follows.

Let $x_{i}^{\left(k\right)}$ and $x_{j}^{\left(k\right)}$ denote the expression quantities for protein *i* and *j* for patient *k*. Protein *i* is the source protein, and protein *j* is a protein in the neighborhood of *i*. The pairwise rank expression representations (PRER) for this patient is defined as:

$$\begin{array}{c} x_{i,j}^{\left(k\right)}=\{\begin{array}{cc} \;1 & \text{if}\;x_{i}^{\left(k\right)}>x_{j}^{\left(k\right)}\\ -1 & \text{otherwise}\; \end{array} \end{array}$$ (4)

$x_{i,j}^{\left(k\right)}=1$ indicates that the molecule *i* is more upregulated with respect to molecule *j* for this patient, whereas $x_{i,j}^{\left(k\right)}=-1$ indicates otherwise. For every *i* in *U* and every *j* in *M*_*i*_, we define a pairwise rank order for the protein pair. If the protein *i*’s phosphorylated state or states are measured, their comparison with *i* is also included. Since the current PPIs do not account for the phosphorylated state (e.g., STATPY705) when we create features for the phosphorylated state of the protein, we use the neighbors of the unphosphorylated (e.g., STAT3) node in the PPI network.

This representation constitutes a nonlinear interaction feature mapping among original features that capture expression dysregulations among interacting proteins. This representation is a nonparametric rank statistics. The rank-based statistics are widely used to obtain a robust statistical analysis. For example, Kendall tau is a robust correlation measure based on pairwise orderings [18]. Since the proposed representation is based on comparisons, it does not require scaling or normalization and will work expression measurements obtained with different experimental technologies.

### Survival prediction

#### Problem description and the survival model

We apply the PRER representation to the survival prediction problem. For each cancer type, the data is of the form, $D={\left\{x^{\left(i\right)},S^{\left(i\right)},\delta^{\left(i\right)}\right\}}_{i=1}^{n}$; *n* is the number of patients. For each patient, **x** is the derived feature vector from protein expression data, *S* is the overall survival time, and *δ* denotes censoring. We use random survival forests for the problem. Random Survival Forest(RSF) [19] is a non-parametric method and is one of the state-of-the-art techniques in survival prediction. It is an ensemble method wherein the base learner is a tree, and each tree is grown on a randomly drawn bootstrap sample. Furthermore, in growing a tree, a randomly selected subset of features is chosen as the candidate features for splitting at each node of the tree. The node is split with the feature among the candidate features that maximize survival difference between child nodes. We used the default values for the rfsrc package [19], where the number of trees is 1000, the number of random splits to consider for each candidate splitting variable is set to 10, and the default splitting rule is log-rank statistics [20, 21].

#### Molecular and clinical data

We test PRER on ten different cancer types in TCGA: ovarian adenocarcinoma (OV), breast invasive carcinoma (BRCA), glioblastoma multiforme (GBM), head and neck squamous cell carcinoma (HNSC), kidney renal clear cell carcinoma (KIRC), lung adenocarcinoma (LUAD), lung squamous cell carcinoma (LUSC), bladder urothelial carcinoma (BLCA), colon adenocarcinoma (COAD), uterine corpus endometrial carcinoma (UCEC). The number of patients for each cancer type ranges from 112 to 841, in total, it is 3253. Details for each cancer are provided in S1 Table. For each cancer type, the number of patients is given at Table A in S1 Text. We obtained TCGA protein expression data and patient survival data from UCSC Cancer Browser (https://genome-cancer.ucsc.edu) (April 11, 2017). The protein expressions are quantified by reverse-phase protein array (RPPA) with a panel of 131 proteins some of which are phosphorylated. For example, RPPA data include STAT3 and STAT3PY705, where STAT3 is Signal Transducer And Activator Of Transcription 3 protein, and STAT3PY705 is the phosphorylation of STAT3 at tyrosine 705 residue. We are able to map all proteins to the PPI network. The obtained RPPA data was already normalized with the replicate-based normalization method [22]. Since PPIs do not represent phosphorylated forms separately, we use the unphosphorylated node when obtaining the neighborhood for the phosphorylated protein.

#### Protein-protein interaction network

We obtained the protein-protein interaction (PPI) network from the InBioMap platform (April 11, 2017). InBioMap specifies a confidence score for each edge, representing the support of the interaction in the literature. Interactions that their confidence scores are lower than 0.1 are filtered out, leading a final network that consists of 17,653 proteins and 625,641 interactions.

## Results and discussion

To assess if PRER representation captures the molecular expression profiles better than the individual protein expression values, we use these representations for survival prediction. We first build two sets of survival prediction models for the 10 cancer types. In building these two sets of models, only the feature representations differ. In the first one, we use the protein expression values as input, which is the typical approach taken in survival prediction. In contrast, in the second one, we use the proposed PRER representation.

Next, we compare our model with two network-based competitor methods from the literature. The first model is by Hofree et al. [7], which uses network propagation to diffuse information on each node over a network. In this paper, Hofree et al. [7] use mutation data for patient stratification. Here, we used the protein expression data, use the same network propagation approach to diffuse the expression values over the network. We input the feature vector that contains the diffused feature values after propagation into the model to predict survival. We implemented this algorithm in R and set network propagation parameter *α* to 0.5 and run the RSF model with default parameters. As the second method, we compare PRER with Reweighted RSF (RRSF) method, proposed by Wang and Liu [13]. RRSF weights the features in random sampling step of RSF model with their topological importance in the PPI network. For RRSF, we use the authors’ implementation.

**Fig 2:**
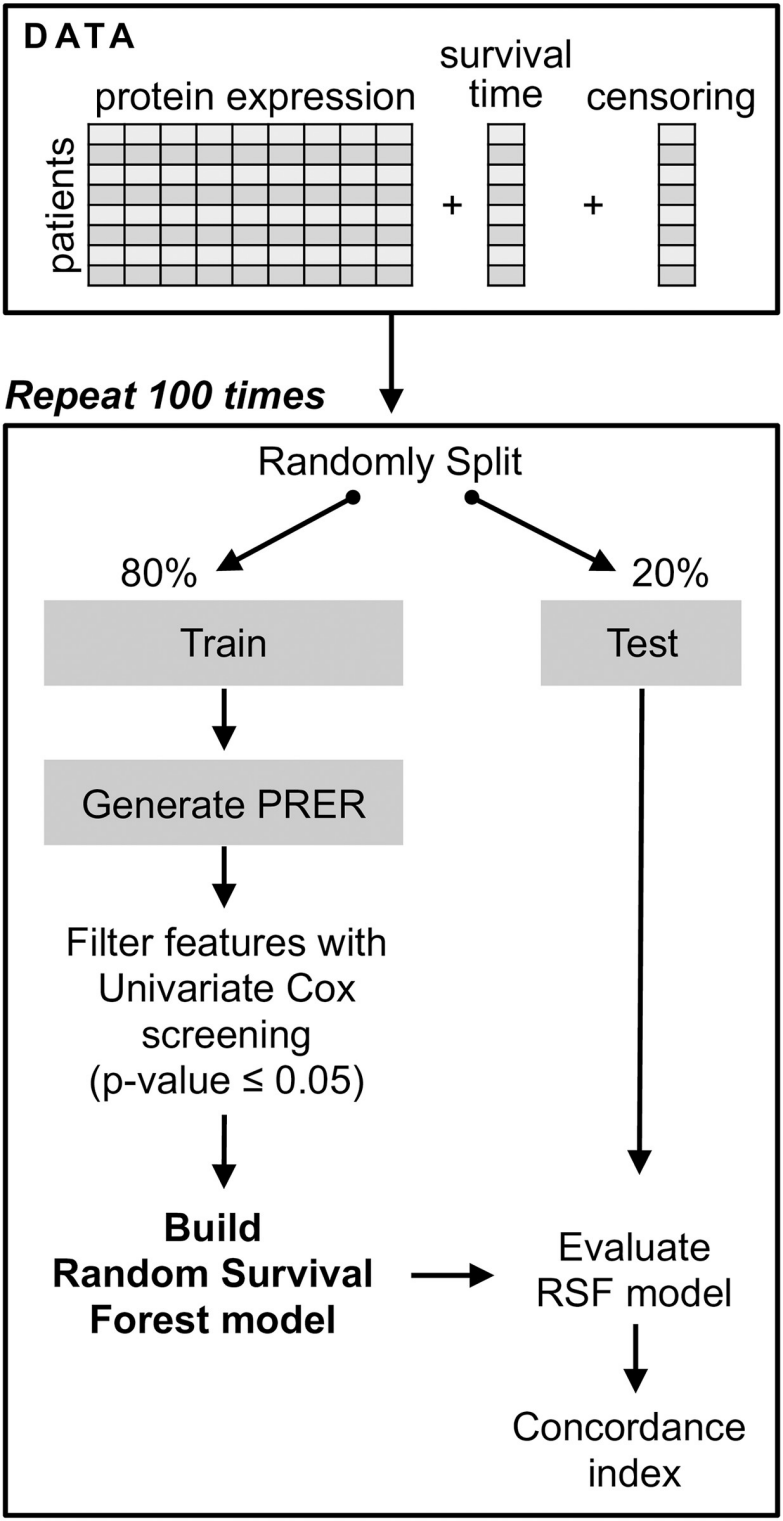
The pipeline for survival prediction. The step that involves generating PRER is skipped when the experiment is run with the alternative method of individual expression values.

For all the models trained, we randomly split the samples into 80% of samples as the training set and 20% as the test set. We repeat this process 100 times leading to 100 different train-test splits and 100 different models. In training each model, we perform a univariate feature selection based on the hazard ratio of the Cox model [23] except for the RRSF model. Features with p-value ≤0.05 are retained for model training. With given random walk parameters in Section 2, and the InBioMap PPI, using 131 proteins in RPPA, PRER produces 1909 dimensional feature vectors for each patient. After applying univariate prefiltering with Cox model [23] to these 1909 dimensional features, we obtained the set of features that go into training, see Table B in S1 Text. Finally, the models’ predictive performances are measured with the Concordance Index (C-index) [24] on the test data. The pipeline of the model training and evaluation is summarized in Fig 2.

### Survival prediction performance of PRER

**Fig 3:**
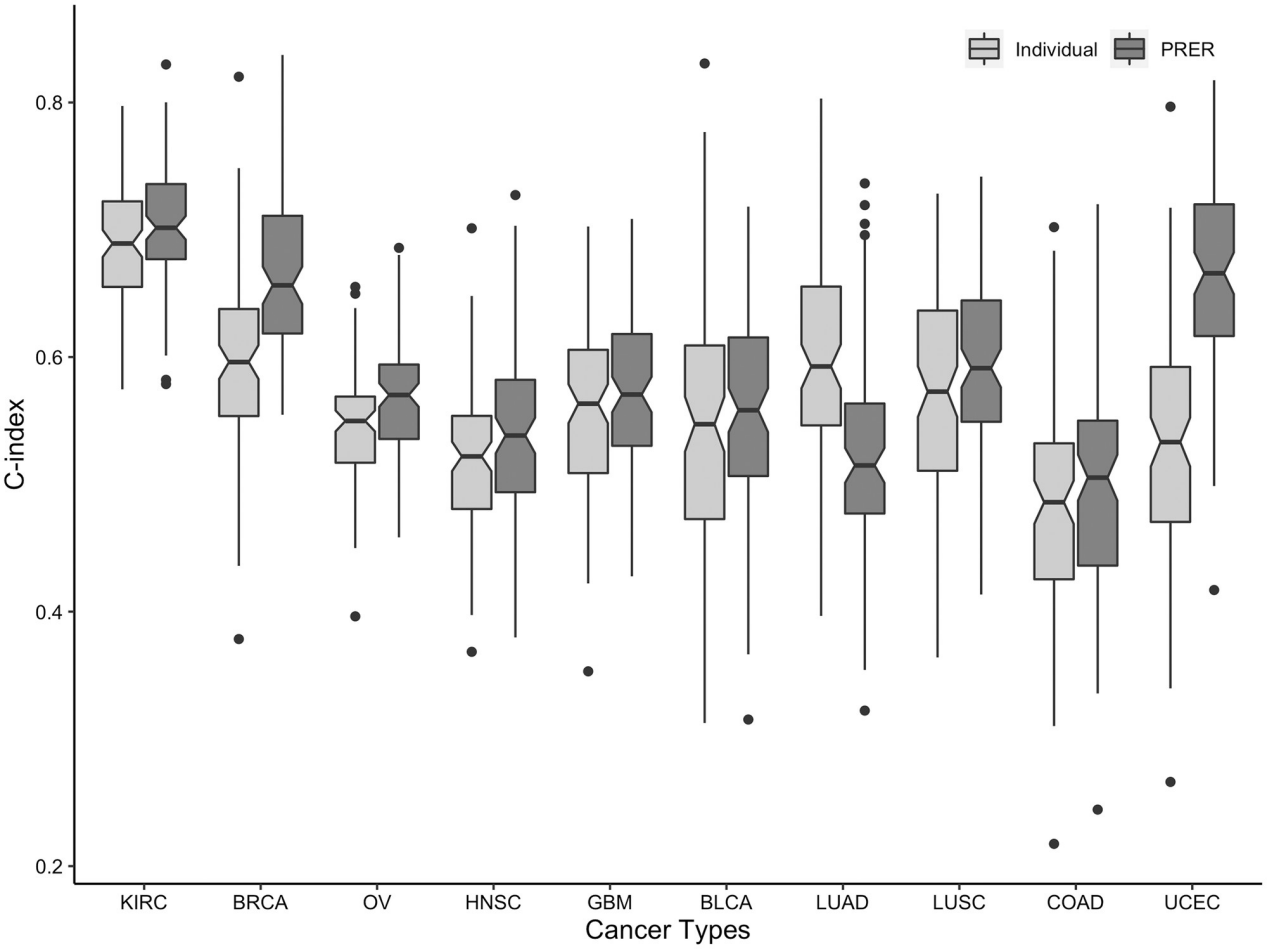
Comparison of RSF model performances that are trained with individual proteins and pairwise ranking representations for different cancer types. The distribution is over 100 models trained that have different random train and test splits. The performances of the models that use the individual expression values as features (Individual) and PRER representation as features (PRER) are compared in each case.

We first compare PRER with the reference models trained with individual features. Fig 3 compares the distribution of C-indices for 100 models trained with the two different feature representations for 10 different cancer types. In 9 of 10 cancer types, PRER representation yields statistically significant improvements (Wilcoxon signed-rank test, BH adjusted p-value <0.05). The C-index quantiles of 100 bootstrap results and corresponding *p*-values are listed in Table C in S1 Text. The best improvements are found in *UCEC*, *BRCA*, *KIRC* and *OV*.

Next, we compare PRER with two other competitive, network propagation by Hofree et al. [7] and RRSF by Wang and Liu [13]. Fig A in S1 Text and Table C in S1 Text detail the result of performance comparisons between the models. To summarize the performance of PRER against the two competitor methods, we present a win/tie/loss table (Table 1). In this table, a win count corresponds to the number of cancer types on which PRER achieves statistically significant performance improvement over its competitor. In contrast, the loss count denotes the number of cancer types on which the compared method achieves statistically significant improvements. If none of the methods can achieve a significant improvement compared to the other, we mark it as a tie. We observe that PRER outperforms both network-based methods in 5 of the cancers, ties with them in 4 cancer types and underperforms only in one cancer. The cancer type where PRER underperforms is LUAD, which we do not observe any improvement with PRER representation (Fig 3).

**Table 1:** Win/Tie/Loss counts of PRER against competing methods. PRER is compared against each model over 100 trained models, where each model is trained on a different train/test split. The comparisons are based on one-sided Wilcoxon signed rank test with BH multiple hypothesis test correction at the significance level of 0.05. The Hofree et al. method is the network propagation algorithm [7]. RRSF stands for reweighted random survival algorithm by Wang and Liu [13].

	vs. Individual	vs. Hofree et al.	vs. RRSF
**PRER**	9 / 0 / 1	5 / 4 / 1	5 / 4 / 1

### Effect of different parameter choices on PRER performance

#### Effect of choice of protein-protein interaction network

To understand the effect of PPI network used, we repeat the experiments on 10 cancers using another network: a PPI network by the IntAct database [25]. This time, we observe statistically significant improvements in 6 out of 10 cancer types. We provide C-index quantiles and Wilcoxon signed-rank test adjusted p-values in Table E in S1 Text. The difference between the two sets of results could be due to the edge density differences of the networks. The InBioMap network contains 17, 653 nodes and 625, 641 edges whereas IntAct database contain 583, 756 edges and 29, 629 nodes. Although the number of nodes is higher in the IntAct PPI, the edge density of InBioMap is four times higher than that of IntAct’s (0.004 vs. 0.001). The edge density is calculated as the number of edges divided by the possible number of edges. This illustrates that PRER performance, as expected, is dependent on the PPI network used.

#### Effect of random walk parameters

In PRER, we define the neighborhood of a protein using random walks. There are several input parameters for the random walk technique which we use: the number of walks, walk length, *p* and *q*. To see their influence on the output of PRER, we conduct runs with various choices of these parameters. Figs C-L in S1 Text contains these parameter sensitivity results. For each cancer, the effect of a parameter is different. For example, as the number of walks or the length of the walk increases, the prediction performance slightly increases for BRCA and the GBM. However, we observe the opposite effect for BLCA and UCEC. For the other cancers, there is no such appreciable effect. In conclusion, the change in *p* and *q* does not drastically change the performance. These hyperparameters can be tuned for each cancer separately for larger patient cohorts. We provide an analysis of each parameter for different cancers in Figs C-L in S1 Text.

#### Effect of the amount of difference between the protein expression levels

PRER representation assigns a binary value to a specific protein pair, either 1 or −1 based on pairwise comparison of the protein expression levels. We experiment with an alternative representation, for which we assign the feature value 0 if the difference between the expression values is less than 10% of the compared neighbor. Otherwise, we assign 1 and -1 based on the comparison. We call this representation as ternary PRER. We compare this ternary feature representation model against the binary feature representation model. We observe no improvements in eight cancer types, but we observe improvement in GBM (p-value = 0.01) and UCEC (p-value = 0.02). The detailed results are presented in Table D in S1 Text and Fig B in S1 Text. What to consider as a meaningful expression difference between pairs of protein probably depends on both the tissue and the protein pairs question. When measurements from matched normal tissue samples are available, the difference threshold could be decided per protein pair and per cancer type. Since we did not have it here, we choose the method with the least assumption and rely on the RSF model to pick up the meaningful features. In future work with richer datasets, this step could be improved.

### Predictive PRER features

**Fig 4:**
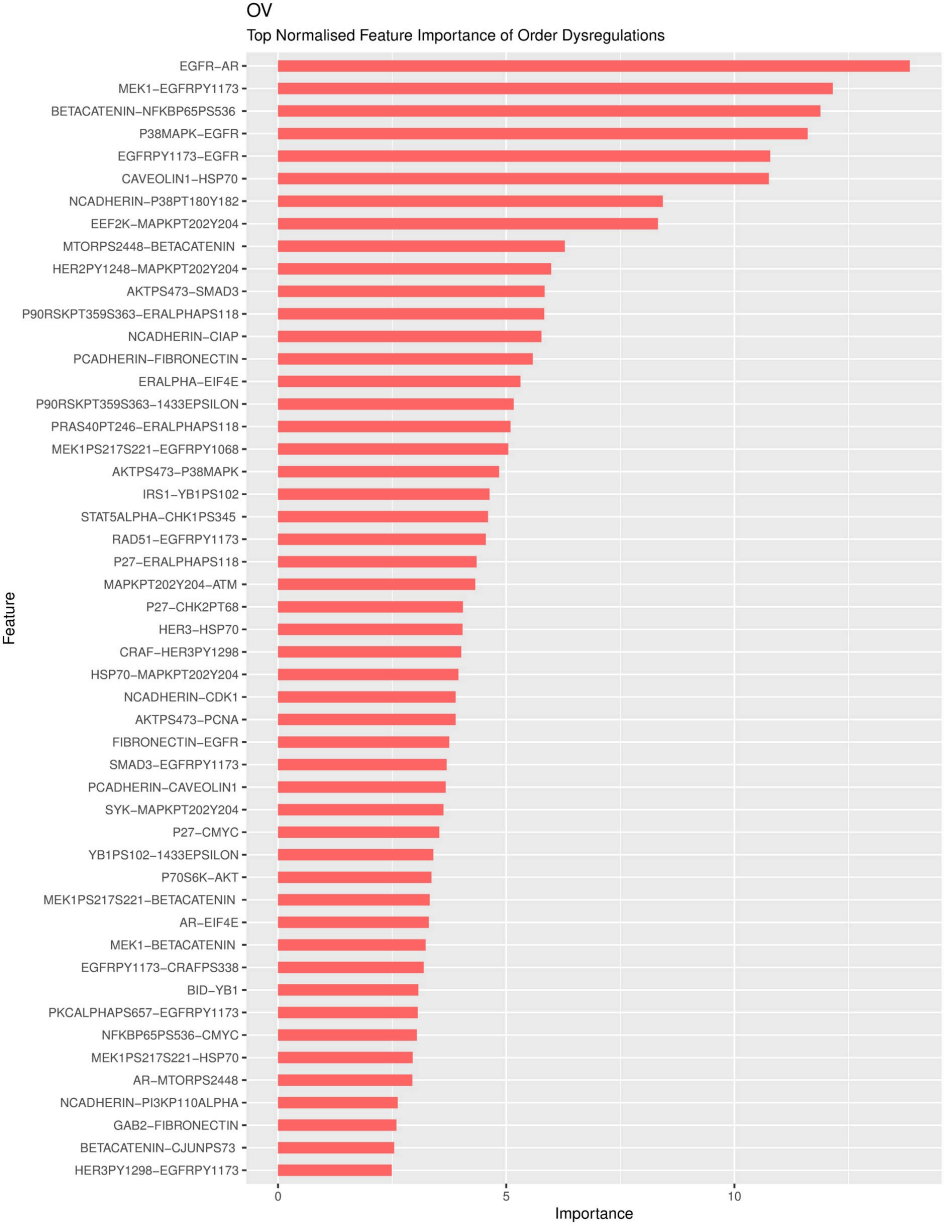
The variable importance of significant pairwise ranking representations for ovarian cancer.

We seek to determine the features ranked as significant in the RSF models trained with PRER features. Note that in these models, pairs of proteins constitute the features. A particular feature’s importance is quantified by the performance difference between the models trained with the original feature vector and the case where the feature vector values are permuted [26]. A significant difference indicates a feature whose absence degrades the model performance. As there are 100 models trained on the repeatedly split data, we calculate the overall feature importance scores over these models as the sum of the scores. We show the normalized feature importance scores for ovarian cancer (OV) in Fig 4. The feature importance scores for other cancer types are available in Figs M-U in S1 Text.

**Fig 5:**
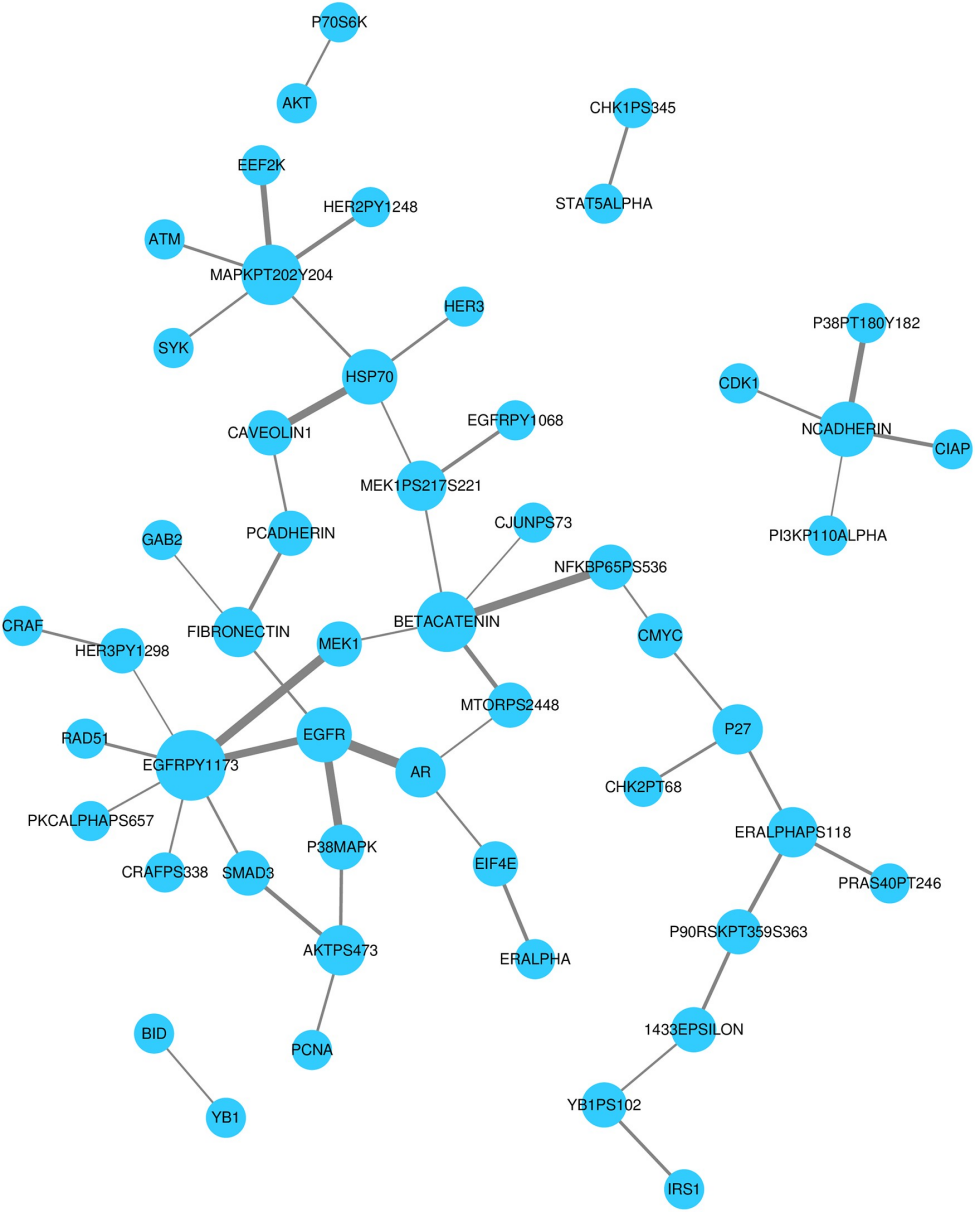
PRER Network for ovarian adenocarcinoma. Nodes represent proteins that appear in the top 50 pairwise ranking representations for ovarian cancer; each edge indicates that two proteins participate in a pairwise rank order feature together. For cases where the expression value pertains to the protein’s phosphorylated state, the ids include the phosphosite’s residue position and the amino acid type.

As shown in Fig 4, some proteins repeatedly show up as partners in the list of important genes. To analyze these relationships, we form a network where the nodes represent proteins that participate in the top 50 PRER features. Edges are formed when a given protein pair is found to be partners in a PRER feature. Fig 5 demonstrates that some proteins emerge as important in many pairs. Several studies support these genes’ association with ovarian cancer. Epidermal growth factor receptor protein (EGFR) and its phosphorylated state EGFRPY1173 are among the top PRER features. EGFR is a receptor protein that receives and transmits signals from the environment to the cell and is the target of drugs in therapies for many cancer types, including ovarian cancer [27, 28]. Marozkina et al. [29] provide results that changes in expression of EGFR may lead to ovarian carcinoma. Others [30–32] also claim that up-regulation of EGFR expression promotes ovarian cancer. Interestingly, Li et al. [33] and Ilekis et al. [31] demonstrate that the levels of EGFR and androgen receptor (AR), which constitute the top feature of PRER in Fig 4, are interacted in ovarian cancer.

Another important protein that participates in important features is Caveolin-1 (CAV1). CAV1 takes on critical roles in cell survival, cell proliferation, cell migration and programmed cell death [34]. An earlier study by Wiechen et al. [35] report that CAV1 is dysregulated among ovarian cancer patients based on microarray expression data. Others also report that CAV1 is dysregulated in different cancer types and its role in chemotherapy resistance [36, 37].

**Fig 6:**
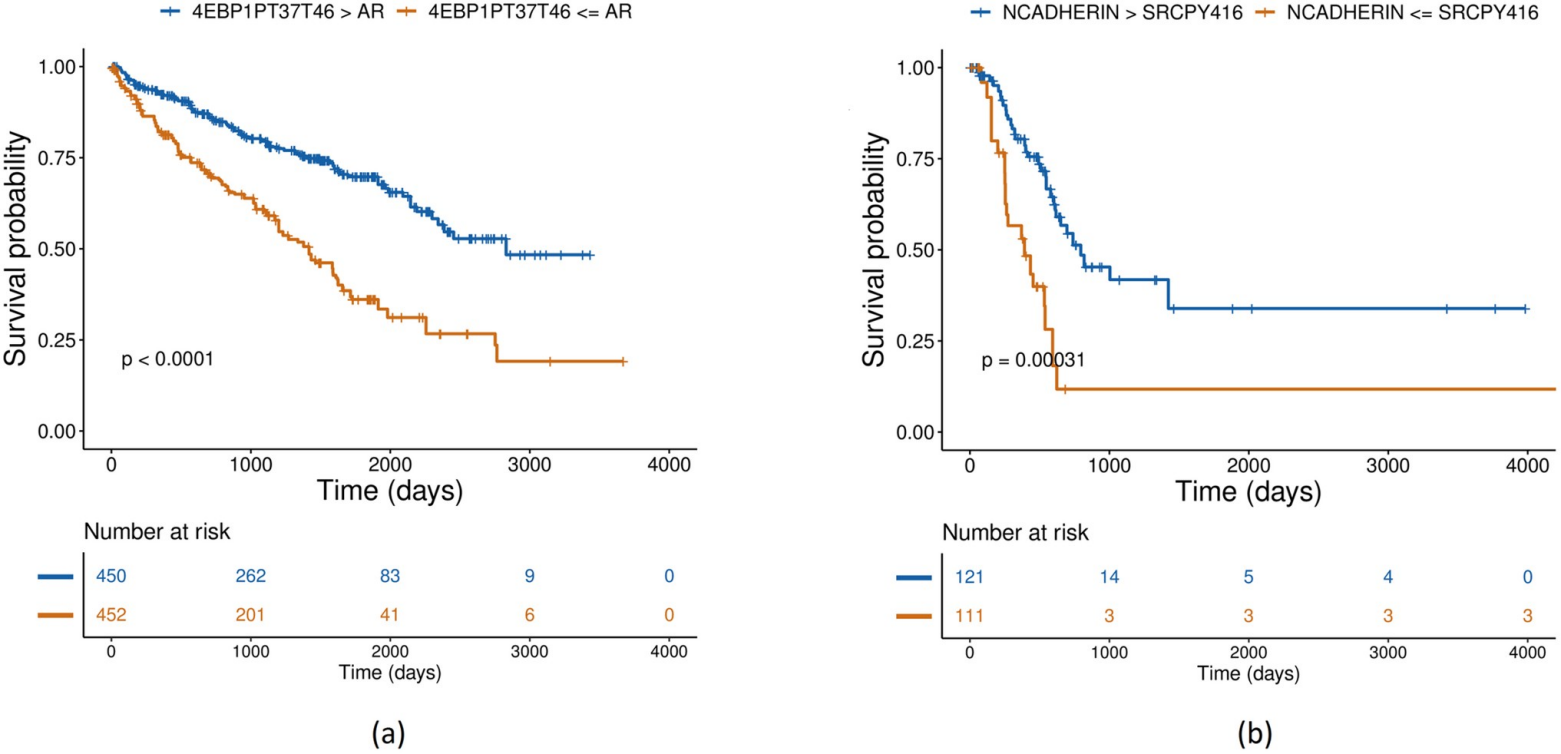
Age and sex adjusted Kaplan-Meier plots for a) KIRC and b) BLCA based on overall survival. Number at risk denotes the number of patients at risk at a given time, and p-value is calculated with the log-rank test.

We list the top-ranked PRER pairs for each cancer in Table 2. We provide the Kaplan-Meier (KM) plots of the top feature for KIRC and BLCA based on overall survival in Fig 6. Based on only one feature, the patients can be grouped into groups that differ significantly in their survival distributions. We provide the KM plots of top-ranked features for the other cancers in Fig AE in S1 Text. The confounding factors such as the age and sex of the patient may influence protein expressions. Therefore, we adjust survival curves for the confounding effects. We also apply log-rank tests to adjusted curves and see that age and sex adjustment gives the same p-values of top PRERs.

**Table 2:** The top PRER feature in each cancer type. The relative expression level of this feature is found to be important in the RSF model. The gene symbols of the corresponding gene are listed. The letter P after the gene symbol indicates that this is the phosphorylated version of the protein. The type of phosphosite and its residue number is provided.

Cancer	Top Rank PRER Protein Pair
BLCA	NCADHERIN-SRCPY416
BRCA	DVL3-P38MAPK
COAD	MRE11-HER3PY1298
GBM	NF2-EGFR
HNSC	ECADHERIN-PAXILLIN
KIRC	4EBP1T37T46-AR
LUAD	XRCC1-CYCLINB1
LUSC	PAXILLIN-YAP
OV	EGFR-AR
UCEC	EIF4E-AKT

We should note that many of the proteins reported in the RPPA assay in the TCGA study are selected due to their relevance to cancer. Thus, these important genes are likely to exhibit the individual importance of PRER partners. Therefore, we suggest an alternative way to exclusively analyze those features which emerge as important in the next section.

### Proteins that emerge as important only in the PRER representation

Since many of the proteins that are in the protein expression data are cancer-related, it is not surprising that they are found to be relevant to cancer. However, proteins that emerge as important in the PRER representation but are not highly ranked in the models trained with individual protein expression values would be interesting. These sets of proteins will reveal proteins whose relative expression states to their neighbors are important as opposed to the expression level being up or down-regulated. To identify these proteins, we first assign a feature importance score to each protein in the PRER representation. As the features are pairs of proteins in the PRER, we calculate the feature importance of a protein by averaging the importance of all corresponding PRER feature importance in which this protein contributes. Let *f*_*i*,*j*_ denotes the features’ importance score of the protein pair *i* and *j*. We calculate the individual feature importance score for molecule *i* as follows:

$$\begin{array}{c} s_{i}=\frac{1}{∥N_{i}∥}\sum_{j\epsilon N_{i}}f_{i,j} \end{array}$$ (5)

where *N*_*i*_ is the set of all pairwise ranking representations that include molecule *i*. *s*_*i*_ represents the average importance of molecule *i* concerning the expression levels of other proteins in its neighborhood. We get the each protein’s rank order based on *s*_*i*_, and a lower rank indicates that the protein is important. Let *r*_*p*_ be the protein’s rank in the models with PRER representation and let *r*_*q*_ be the rank order in the models trained with individual protein expressions. To find the proteins whose ranks are low in the models trained with protein expression but are highly ranked in the PRER models, we measure the differences of feature ranks, *r*_*q*_ − *r*_*p*_. Table 3 lists the top 10 proteins in each cancer based on this *r*_*q*_ − *r*_*p*_ difference. We provide the full list of the ranks and differences in S1 Table. A large positive difference points to those proteins for which the relative expression relations of this protein to other proteins in its neighborhood carry prognostic value as opposed to its expression value.

**Table 3:** Top-10 rank differentiated features in each cancer with PRER.

**BLCA**	**BRCA**	**COAD**	**GBM**	**HNSC**
YB1	YB1PS102	RAD50	EGFR	YAP
SRCPY416	STAT5ALPHA	MTORPS2448	PI3KP110ALPHA	STATHMIN
JNKPT183Y185	CKIT	MRE11	PDK1PS241	SMAD4
YB1PS102	CHK2PT68	NF2	PTEN	LKB1
RAD51	PTEN	TUBERIN	PRAS40PT246	NCADHERIN
NCADHERIN	YB1	NCADHERIN	MRE11	PKCDELTAPS664
STATHMIN	CYCLINB1	MIG6	NFKBP65PS536	P27
XRCC1	EEF2	STAT5ALPHA	P38PT180Y182	PDK1PS241
NF2	YAPPS127	HER3PY1298	SRCPY416	P38MAPK
TUBERIN	P53	PI3KP110ALPHA	NOTCH1	PKCALPHAPS657
**KIRC**	**LUAD**	**LUSC**	**OV**	**UCEC**
SMAD1	XRCC1	YAP	EGFR	ASNS
DJ1	YB1	P38PT180Y182	PRAS40PT246	PRAS40PT246
NF2	ASNS	LKB1	YB1	STATHMIN
KU80	STAT3PY705	P70S6K	PCADHERIN	P27PT157
GSK3ALPHABETA	PTEN	RAD50	RAD51	RAD51
4EBP1PS65	YAPPS127	MTOR	SMAD3	SMAD4
PR	YAP	XRCC1	HER3	MIG6
EEF2K	RAD50	SMAD4	PKCALPHAPS657	P90RSKPT359S363
STATHMIN	STATHMIN	BIM	CIAP	PCADHERIN
CIAP	EGFR	ERALPHAPS118	CHK2PT68	YB1PS102

We analyze a subset of the proteins in Table 3. The relevance of the relative expressions of proteins for survival is not reported. Some proteins known to be cancer drivers and perturbed in cancers such as PTEN or EGFR do not rank high in the model wherein the protein expression data is used as input, but in PRER models, they emerge as important. For example, EGFR is ranked as the 16^*th*^ most important feature for ovarian cancer in the models trained with PRER, while it is ranked as the least significant one in the models trained with individual expressions only. Similarly, for GBM, EGFR is ranked as the least significant protein in individual expression models, while it is ranked as the 13^*th*^ most significant feature in PRER. Thus, the PRER models actually highlight that the dysregulation of EGFR expression with respect to its neighbors is an important feature. Below we mention other interesting observations in Table 3.

STAT3PY705 (STAT3 phosphorylation at tyrosine 705), phosphorylated state of STAT3 (Signal Transducer and Activator of Transcription 3) protein, and STAT5ALPHA (Signal Transducer And Activator Of Transcription 5A) also appear in multiple cancer types. While we observe STAT3PY705 as significant in LUAD, STAT5ALPHA appears in BRCA and COAD in Table 3. Activation in the STAT family is reported, especially for STAT3 and STAT5, in several cancer cell lines including head and neck, breast, kidney, ovarian and colorectal [38–41].

YAPPS127 and YAP proteins, which are encoded with the YAP1 (Yes-associated protein 1) gene, are found important in BRCA, HNSC, LUAD, and LUSC cancer types in Table 3. YAP1 is involved in the Hippo signaling pathway that is associated with the growth, development and repair of the cells, and influences the survival of multiple cancers [42]. Poma et al. [43] reports that 17 genes (out of 32) in the Hippo pathway have effects on survival in more than 20 different cancer types and conclude that YAP1 is relevant to the survival of head and neck carcinoma, hepatocellular, lung adenocarcinoma, gastric, pancreatic and colorectal cancers. Further, other studies also suggest that survival for different cancer types is associated with the expression level of YAP1 and its differential expression is considered as a biomarker for bladder urothelial carcinoma (BLCA) [44], breast invasive carcinoma (BRCA) [45–48], ovarian serous cystadenocarcinoma (OV) [49, 50].

The upregulation of STATHMIN is linked with poor survival for primary HNSC [51], and Kouzu et al. [52] suggest that it may be used for the prognosis and a therapeutic target for oral squamous-cell carcinoma, which is the most common type of HNSC. Likewise, the upregulation of STATHMIN is significantly correlated with several cancer types such as LUAD [53], gastric cancer [54, 55], UCEC [56], OV [57] and BRCA [58–60].

YB1 and its phosphorylated state YB1PS102 show correlation with many genes that have functions such as resistance to drugs, transcription and translation of cancerous cells [61]. Although the down-regulation of YB1 is found to be correlated with the reduction in progression, development of cell and programmed cell death at various cancer cells such as breast, colon, lung, prostate and pediatric glioblastoma by some studies [62, 63], there are studies [64–68] showing the association between overexpression of YB1 and different cancer types such as breast, colorectal, glioblastoma, lung, liver, ovarian cancers.

## Conclusion and future work

Predicting patient survival using omics profiles still remains to be a challenge for cancer. If achieved, it can guide the decision-making process for choosing optimal treatment and surveillance strategies among alternative options. Typically, clinical or pathological features such as the patient’s age, tumor stage or grade are employed to predict the clinical outcomes. With the advent of high-throughput technologies, molecular descriptions of the tumors for a large number of patients across many cancer types have become available. However, it remains a significant challenge to use this data due to the high level of genomic heterogeneity among patients. This study proposes a novel patient representation method, PRER. PRER is based on a pairwise comparison of a protein’s expression values with the other proteins in its neighborhood on the PPI network. In this way, the relative expression level patterns with respect to the proteins in their neighborhood can be captured.

We showcase PRER in survival prediction for ten different cancer types. PRER with Random Survival Forest (RSF) model achieves significant improvements compared to the models with individual expression values in 9 of the 10 cancers. The only cancer type that PRER underperforms is LUAD. It is the only cancer type for which the number of patients available is small and the ratio of the censored patient to deceased patient is high (See Table A in S1 Text), which might have resulted in the performance degradation of PRER.

We also suggest ways to delineate the importance of proteins not through their individual up or down-regulation patterns but their relative expressions compared to their neighbors. Such an analysis can provide fundamental mechanistic insights into the studied diseases. The identified pairwise relations could also help design therapies to regulate the pairwise interaction as opposed to regulating the expression level of one protein.

One limitation of the current study is that we use a generic protein-protein interaction network, disregarding whether the protein is expressed in the tissue of a given cancer type. As tissue specific reliable PPI networks become available, we can improve the survival models by incorporating these. A second limitation is that in PRER, we compare the protein expression levels and assign the feature value 1 or -1 based on this difference. We also experiment with a ternary representation where we require this difference to be 10% of the expression level of the protein neighbor compared. These are, of course, ad-hoc choices. What constitutes a large enough difference depends on the tissue type and the protein pair in question. For certain pairs, large differences could be tolerated due to regulatory feedback mechanisms among genes or proteins performing similar functions, while for certain pairs of proteins, minuscule differences can have a large impact on the cellular processes. The ideal scenario would be to decide this threshold based on expression values of the same protein pair in matched normal samples. Since we do not have data for matched normal tissue, here we choose the method with the least number of assumptions and rely on the RSF model to pick up the predictive features. In future work, with the increasing availability of richer datasets, this step can be improved.

In this work, since we aim to assess the PRER representation power, we only use features related to expression. The survival model can be further improved with other clinical features such as age, duration of the follow-up, and cancer stage. PRER representation can be used with other data types, such as mRNA expression and DNA methylation. However, we should note that the number of features increases quadratically with the size of the original features as each feature is compared with its neighboring proteins. In this case, a more stringent feature filtering step, reducing the number of neighbors or a regularized prediction model will be helpful.

## Supporting information

S1 Text Supplementary file for PRER. All supporting tables and figures mentioned in the manuscript.
(PDF)

S1 Table Feature rank differences in each cancer with PRER. (XLSX)
