# Supplementary material for: PRER: A patient representation with pairwise relative expression of proteins on biological networks
Source: PLoS Comput Biol. 2021 May 26;17(5):e1008998. doi: 10.1371/journal.pcbi.1008998 (PMC8238204; doi:10.1371/journal.pcbi.1008998)
Supplement: S1 Text — All supporting tables and figures mentioned in the manuscript. (PDF) [file pcbi.1008998.s001.pdf]

Supplementary Material for PRER: A Patient Representation with Pairwise Relative Expressions on Biological Networks

Halil İbrahim Kuru   Mustafa Buyukozkan   Oznur Tastan

1 Supplementary Tables

1.1 Clinical and Molecular Patient Data

Number of Patients		
	censored	deceased
KIRC	297	156
OV	194	213
BRCA	628	99
LUAD	147	72
LUSC	106	78
COAD	260	60
HNSC	98	114
BLCA	70	52
UCEC	347	50
GBM	73	139

Table A: Number of censored and deceased patients for each cancer type.

**Cancer Type**	**Average number of PRER features remain after Cox-fitering**
**BRCA**	118
**BLCA**	336
**COAD**	114
**HNSC**	163
**GBM**	133
**KIRC**	778
**LUAD**	90
**LUSC**	189
**OV**	202
**UCEC**	312

Table B: The average number of PRER features that pass the Cox filtering for each cancer.

1.2 Prediction Performance

**Random Survival Forest C-indices**							
	C-index					p-value	BH adjusted p-value
	1st	2nd	median	3rd	4th		
KIRC	0.57	0.66	0.69	0.72	0.80		
KIRC-prer	0.58	0.68	**0.70**	0.74	0.83	**7.1×10^{-11}**	**2.4×10^{-10}**
KIRC-rrsf	0.48	0.61	0.64	0.68	0.74	1	1
KIRC-hofree	0.56	0.67	**0.70**	0.73	0.80	**7.8×10^{-4}**	**1.9×10^{-3}**
OV	0.40	0.52	0.55	0.57	0.66		
OV-prer	0.46	0.54	**0.57**	0.59	0.69	**5.4×10^{-7}**	**1.3×10^{-6}**
OV-rrsf	0.42	0.50	0.52	0.56	0.66	0.99	1
OV-hofree	0.41	0.53	**0.56**	0.59	0.68	**2.7×10^{-4}**	**9.1×10^{-4}**
BRCA	0.38	0.55	0.60	0.64	0.82		
BRCA-prer	0.55	0.62	**0.66**	0.71	0.84	**9.3×10^{-15}**	**4.7×10^{-14}**
BRCA-rrsf	0.41	0.57	**0.61**	0.66	0.76	0.18	0.36
BRCA-hofree	0.39	0.54	0.59	0.63	0.77	1	1
LUSC	0.36	0.51	0.57	0.64	0.73		
LUSC-prer	0.41	0.55	**0.59**	0.64	0.74	**4.1×10^{-3}**	**6.8×10^{-3}**
LUSC-rrsf	0.42	0.54	**0.60**	0.64	0.78	$5.2x10^{-2}$	0.13
LUSC-hofree	0.34	0.55	**0.60**	0.66	0.75	**1.8×10^{-4}**	**9×10^{-4}**
COAD	0.22	0.43	0.49	0.53	0.70		
COAD-prer	0.24	0.44	**0.51**	0.55	0.72	**0.02**	**0.02**
COAD-rrsf	0.28	0.47	**0.53**	0.61	0.75	**1.1×10^{-4}**	**3.8×10^{-4}**
COAD-hofree	0.29	0.45	**0.53**	0.58	0.72	**2.8×10^{-3}**	**4.7×10^{-3}**
HNSC	0.37	0.48	0.52	0.55	0.70		
HNSC-prer	0.38	0.49	**0.54**	0.58	0.73	**1.3×10^{-4}**	**2.7×10^{-4}**
HNSC-rrsf	0.38	0.51	**0.55**	0.60	0.68	**6.8×10^{-5}**	**3.4×10^{-4}**
HNSC-hofree	0.36	0.50	**0.54**	0.57	0.76	**1.2×10^{-3}**	**2.5×10^{-3}**
UCEC	0.27	0.47	0.53	0.59	0.80		
UCEC-prer	0.42	0.62	**0.67**	0.72	0.85	**8.1×10^{-18}**	**8.1×10^{-17}**
UCEC-rrsf	0.35	0.56	**0.61**	0.66	0.88	**1.3×10^{-6}**	**1.3×10^{-5}**
UCEC-hofree	0.30	0.52	**0.56**	0.63	0.73	**5.8×10^{-5}**	**5.8×10^{-4}**
GBM	0.35	0.51	0.56	0.61	0.70		
GBM-prer	0.43	0.53	**0.57**	0.62	0.71	**0.02**	**0.03**
GBM-rrsf	0.41	0.52	0.55	0.60	0.69	0.58	0.97
GBM-hofree	0.36	0.52	**0.56**	0.61	0.75	0.13	0.19
BLCA	0.31	0.47	0.55	0.61	0.83		
BLCA-prer	0.32	0.51	**0.56**	0.62	0.72	**0.02**	**0.03**
BLCA-rrsf	0.29	0.46	0.52	0.58	0.69	0.90	1
BLCA-hofree	0.24	0.50	0.54	0.59	0.70	0.86	1
LUAD	0.40	0.55	0.59	0.66	0.80		
LUAD-prer	0.32	0.48	0.51	0.56	0.74	1	1
LUAD-rrsf	0.38	0.53	**0.60**	0.65	0.77	0.75	1
LUAD-hofree	0.34	0.50	0.56	0.62	0.78	1	1

Table C: The comparison of RFSs models' performances that are trained with different feature representations for different cancer types. The methods are compared at the mean with four different quartile values of the 100 repeated runs, where each run is conducted with a random split of train and test data. The p-values of the one-tailed Wilcoxon signed-rank test of comparing the each model with Individual model is listed. The BH column lists the adjusted p-value after Benjamini and Hochberg correction for multiple hypothesis test correction [Benjamini and Hochberg(1995)].

1.3 Performance Comparison of proposed PRER representation with Ternary PRER

Cancer Type	p-value (compared to PRER)	BH adjusted p-value (compared to PRER)
**BRCA**	0.9	1
**BLCA**	1	1
**COAD**	0.07	0.23
**HNSC**	0.21	0.41
**GBM**	0.01	0.08
**KIRC**	0.99	1
**LUAD**	0.80	1
**LUSC**	0.99	1
**OV**	0.11	0.27
**UCEC**	0.02	0.08

Table D: Comparison of RSF model performances that are trained with two PRER settings for each cancer type. The performance of the proposed PRER repsesentation (PRER) is compared with the PRER representation with three value as features (Ternary PRER) in each case. Wilcoxon signed-rank test is applied to calculate p-value for of each setting with respect to PRER representation. The BH column lists the adjusted p-value after Benjamini and Hochberg correction for multiple hypothesis test correction [Benjamini and Hochberg (1995)].

1.4 Prediction Performance with IntAct PPI Network

**Random Survival Forest C-indices**							
	C-index					p-value	BH adjusted p-value
	1st	2nd	median	3rd	4th		
KIRC	0.57	0.66	0.69	0.72	0.80	**1.1×10^{-8}**	**3.6×10^{-8}**
KIRC-prer	0.56	0.68	**0.70**	0.73	0.83		
OV	0.40	0.52	0.55	0.57	0.66	**0.21**	0.30
OV-prer	0.44	0.52	0.55	0.58	0.66		
BRCA	0.38	0.55	0.60	0.64	0.82	**8.3×10^{-8}**	**1.7×10^{-7}**
BRCA-prer	0.47	0.58	**0.64**	0.70	0.80		
LUSC	0.36	0.51	0.57	0.64	0.73	**5.3×10^{-9}**	**2.7×10^{-8}**
LUSC-prer	0.44	0.58	**0.63**	0.68	0.78		
COAD	0.22	0.43	0.49	0.53	0.70	**1.2×10^{-4}**	**2.0×10^{-4}**
COAD-prer	0.34	0.46	**0.52**	0.57	0.70		
HNSC	0.37	0.48	0.52	0.55	0.70	**3.9×10^{-8}**	**9.9×10^{-8}**
HNSC-prer	0.38	0.52	**0.56**	0.60	0.71		
UCEC	0.27	0.47	0.53	0.59	0.80	**3.6×10^{-17}**	**3.6×10^{-16}**
UCEC-prer	0.46	0.61	**0.66**	0.70	0.86		
GBM	0.35	0.51	0.56	0.61	0.70	**0.47**	0.59
GBM-prer	0.43	0.53	**0.57**	0.62	0.71		
BLCA	0.31	0.47	0.55	0.61	0.83	**0.99**	1
BLCA-prer	0.27	0.45	**0.51**	0.57	0.77		
LUAD	0.40	0.55	0.59	0.66	0.80	1	1
LUAD-prer	0.23	0.42	0.48	0.54	0.81		

Table E: The comparison of RFSs models' performances that are trained with individual features and pairwise ranking representations (PRER) in IntAct PPI for different cancer types. The methods are compared at the mean with four different quartile values of the 100 repeated runs, where each run is conducted with a random split of train and test data. The p-values of the one-tailed Wilcoxon signed-rank test of comparing the two models is listed. The BH column lists the adjusted p-value after Benjamini and Hochberg correction for multiple hypothesis test correction[Benjamini and Hochberg(1995)].

2 Supplementary Figures

2.1 PRER Compared to Alternative Methods

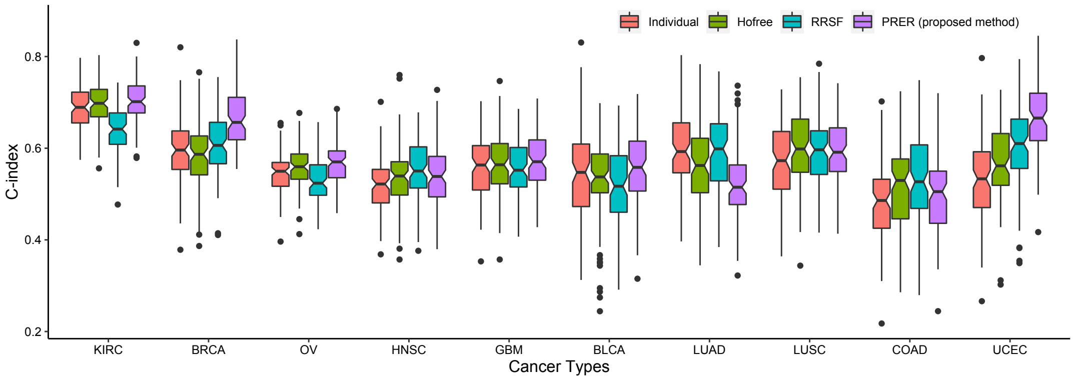

Figure A: Comparison of different RSF prediction models. Hofree: a model that uses network propagated protein expressions as features, Individual: a model that is trained with individual protein expressions, PRER: the proposed PRER representation, RRSF: a model that is proposed by Wang et al. 2018.

2.2 Performance Comparisons of Two PRER Settings

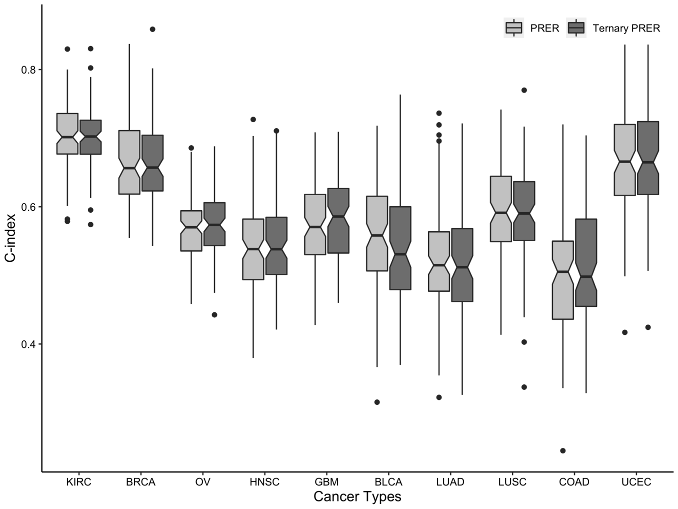

Figure B: Comparison of different models trained with different PRER settings for each cancer type. PRER: the proposed PRER representation (PRER), Ternary PRER: PRER representation with three different assignment of feature values (-1, 0, 1). The box-plots shows the distribution over 100 models that are trained on different random train/test splits. The models are compared statistically in Supplementary Table D.

2.3 The Effect of Random Walk Parameters on Performance

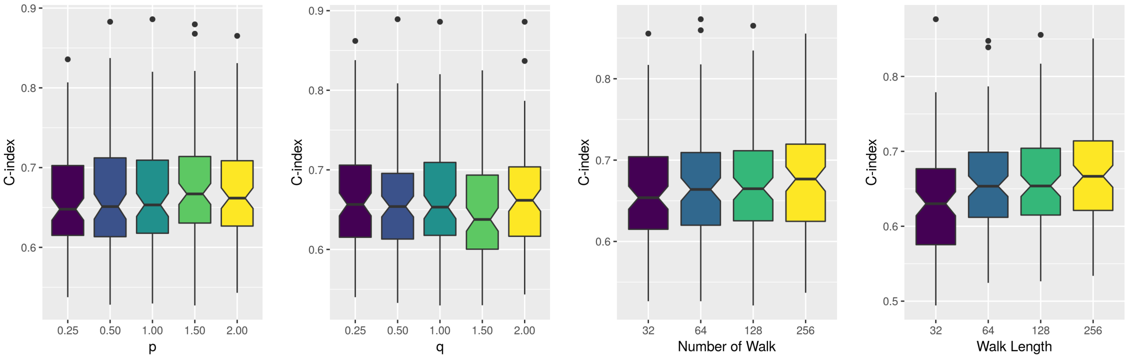

(a) (b) (c) (d)

Figure C: Parameter sensitivity for BRCA. At each subfigure, corresponding parameter is varied and all other parameters are fixed.

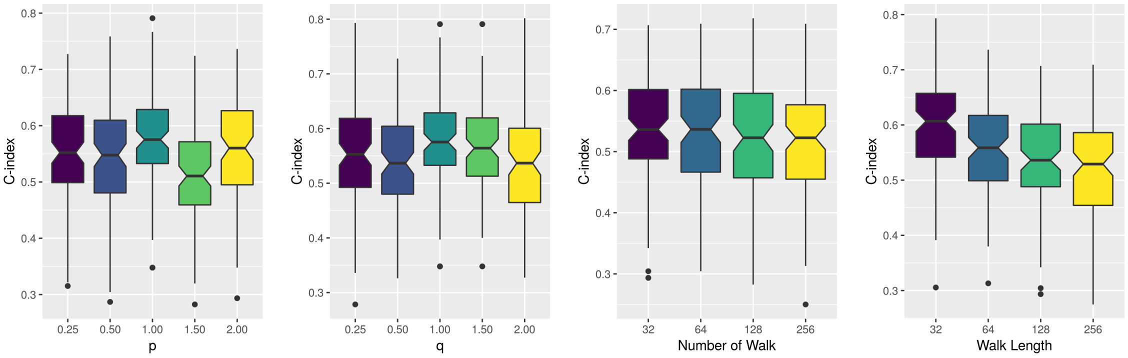

(a) (b) (c) (d)

Figure D: Parameter sensitivity for BLCA. At each subfigure, corresponding parameter is varied and all other parameters are fixed.

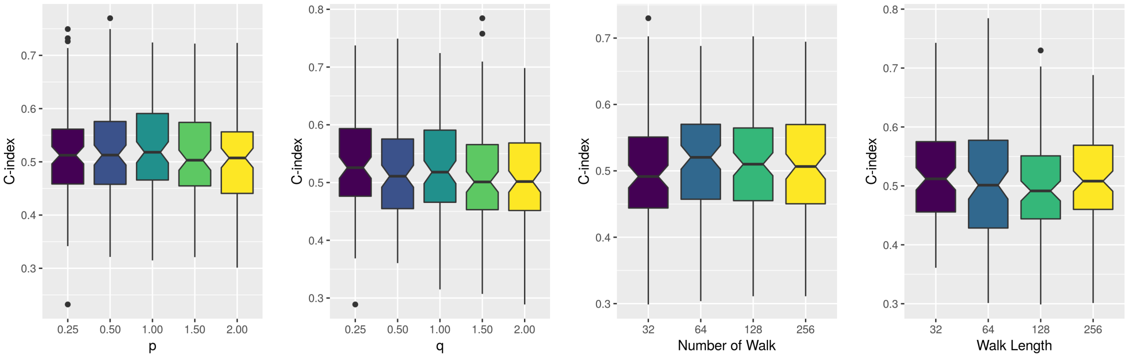

(a) (b) (c) (d)

Figure E: Parameter sensitivity for COAD. At each subfigure, corresponding parameter is varied and all other parameters are fixed.

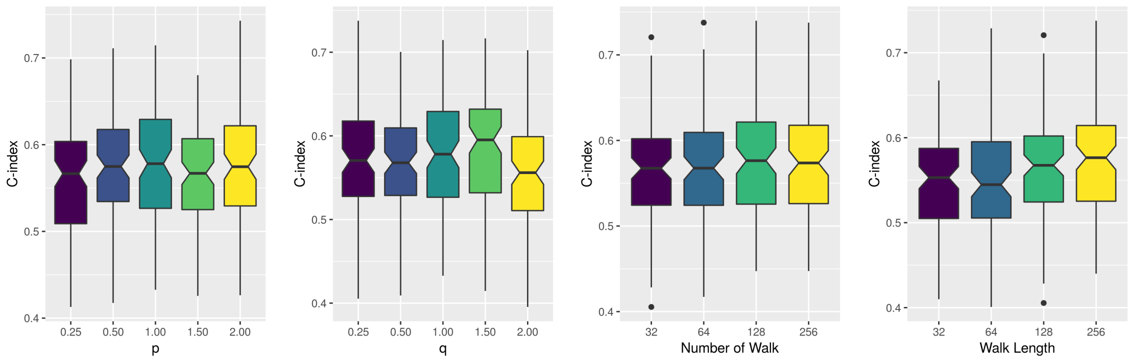

(a) **(b)** **(c)** **(d)**

Figure F: Parameter sensitivity for GBM. At each subfigure, corresponding parameter is varied and all other parameters are fixed.

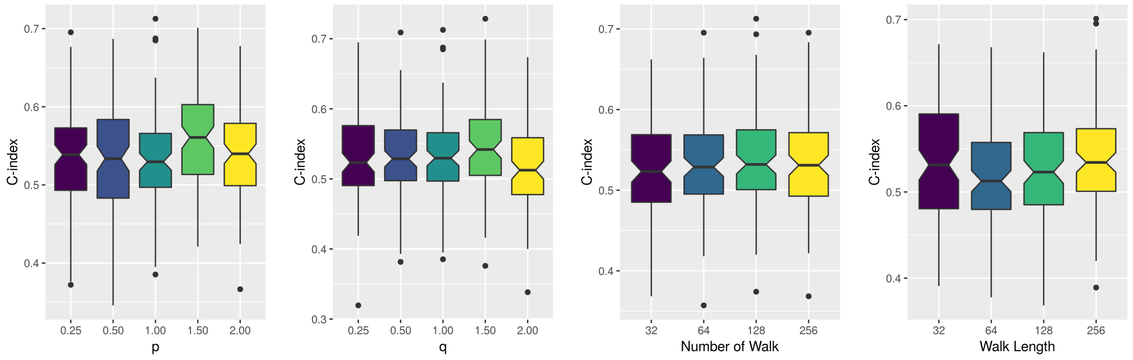

(a) **(b)** **(c)** **(d)**

Figure G: Parameter sensitivity for HNSC. At each subfigure, corresponding parameter is varied and all other parameters are fixed.

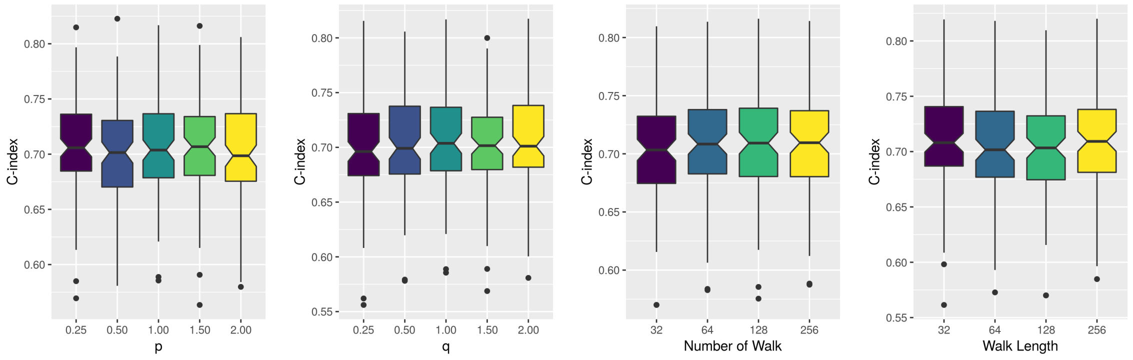

(a) **(b)** **(c)** **(d)**

Figure H: Parameter sensitivity for KIRC. At each subfigure, corresponding parameter is varied and all other parameters are fixed.

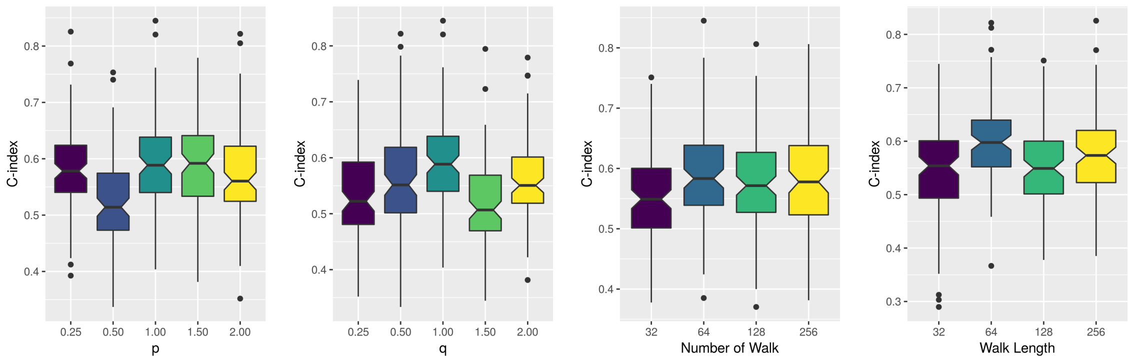

(a) **(b)** **(c)** **(d)**

Figure I: Parameter sensitivity for LUAD. At each subfigure, corresponding parameter is varied and all other parameters are fixed.

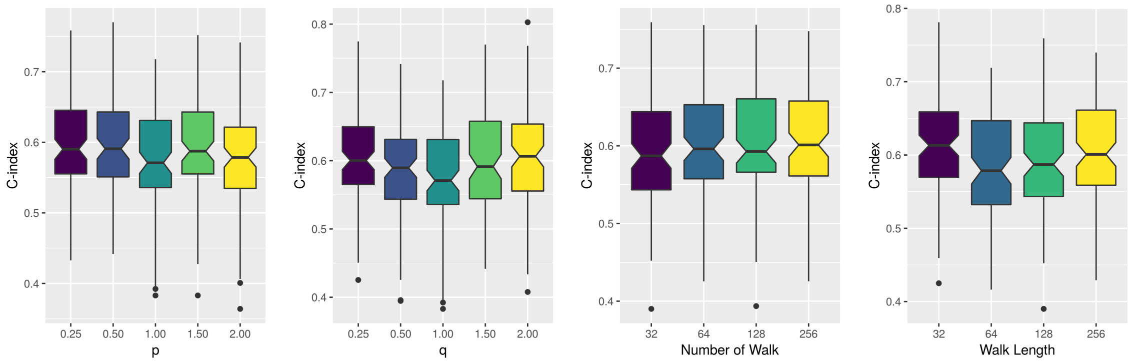

(a) **(b)** **(c)** **(d)**

Figure J: Parameter sensitivity for LUSC. At each subfigure, corresponding parameter is varied and all other parameters are fixed.

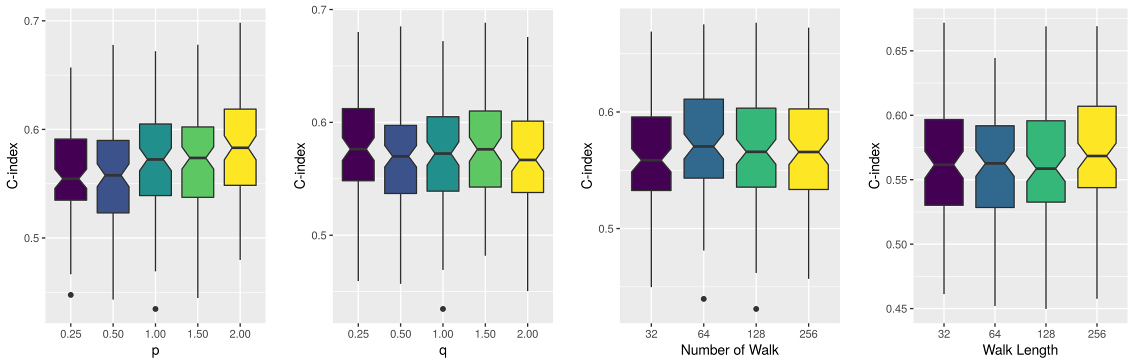

(a) **(b)** **(c)** **(d)**

Figure K: Parameter sensitivity for OV. At each subfigure, corresponding parameter is varied and all other parameters are fixed.

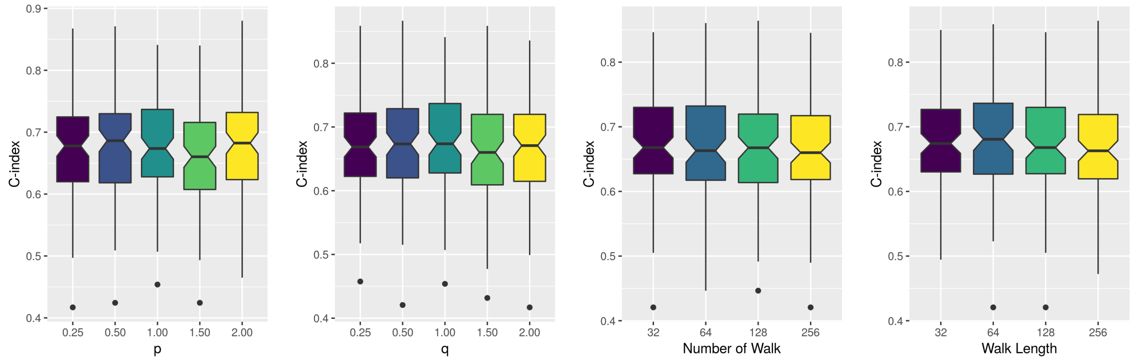

(a) (b) (c) (d)

Figure L: Parameter sensitivity for UCEC. At each subfigure, corresponding parameter is varied and all other parameters are fixed.

2.4 Important PRER Features

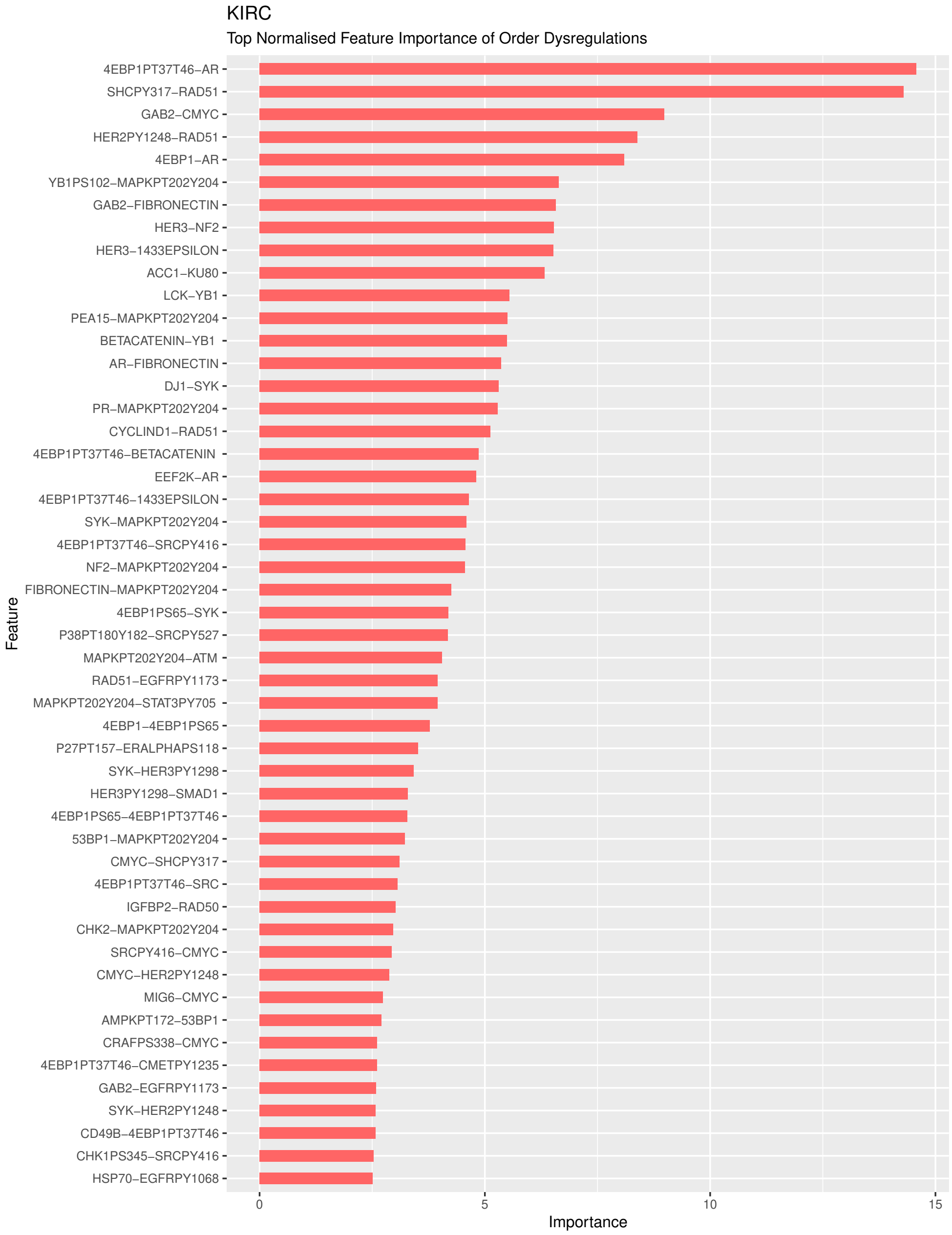

Figure M: Variable importance of significant pairwise ranking embeddings for kidney renal clear cell carcinoma.

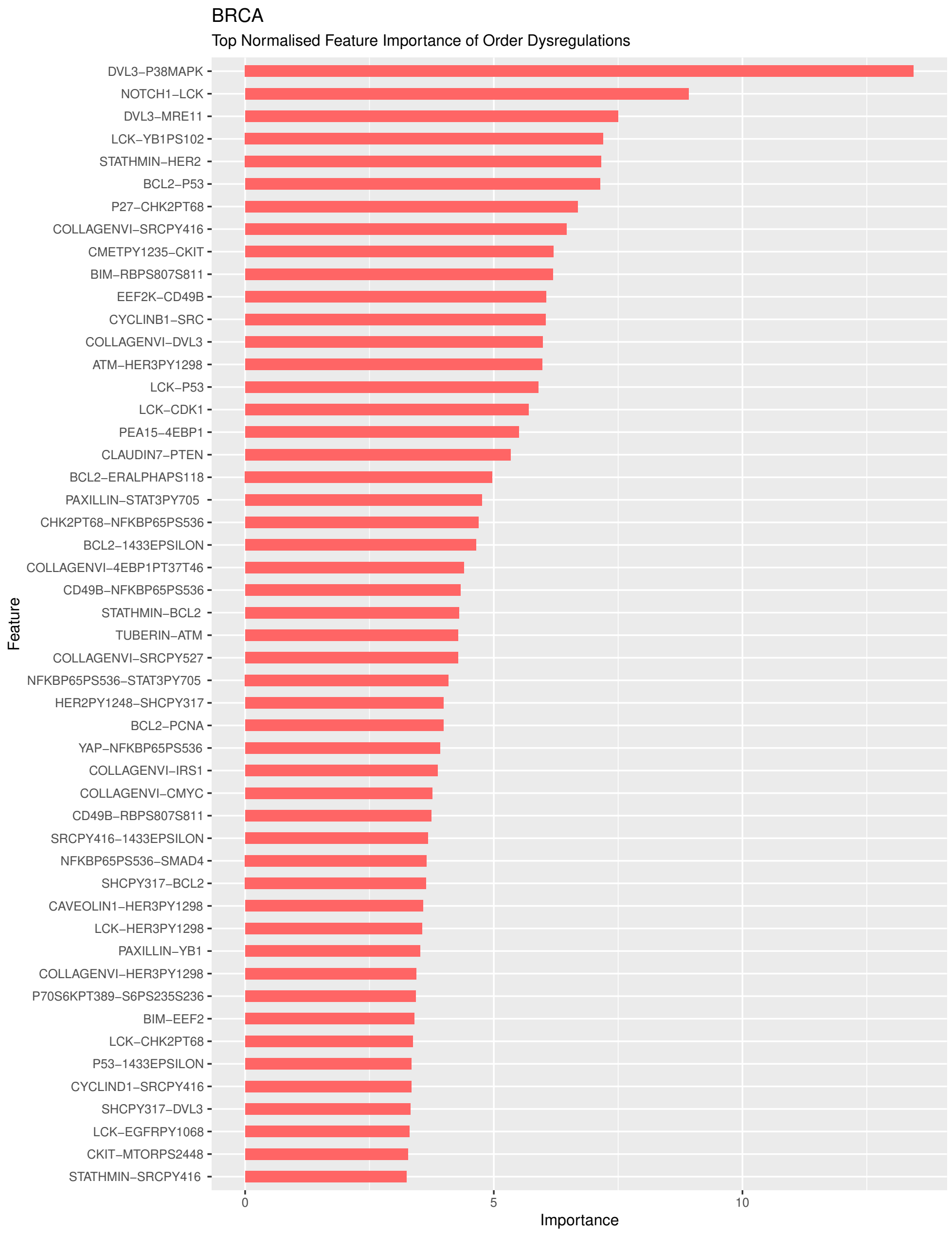

Figure N: Variable importance of significant pairwise ranking embeddings for breast invasive carcinoma.

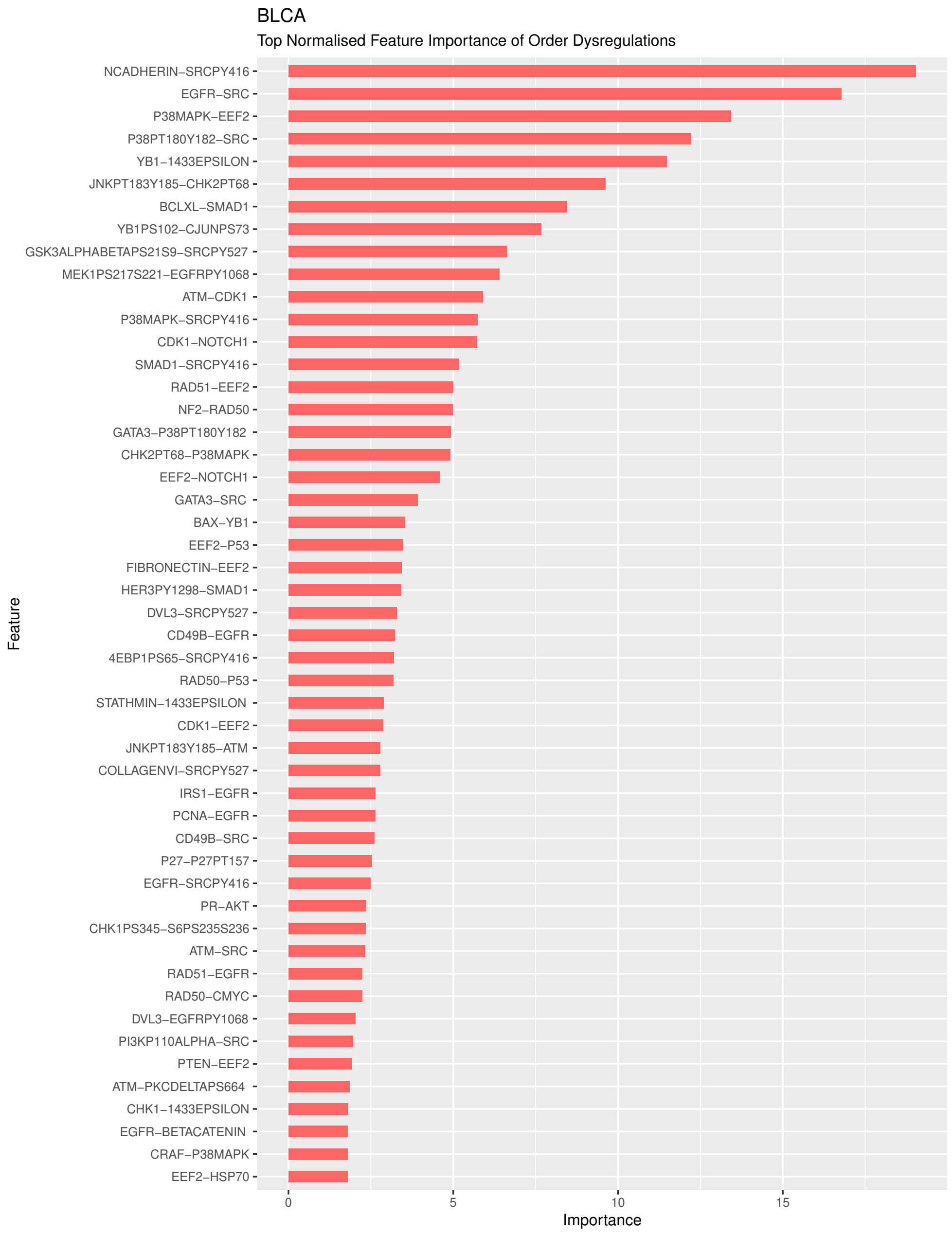

Figure O: Variable importance of significant pairwise ranking embeddings for bladder urothelial carcinoma.

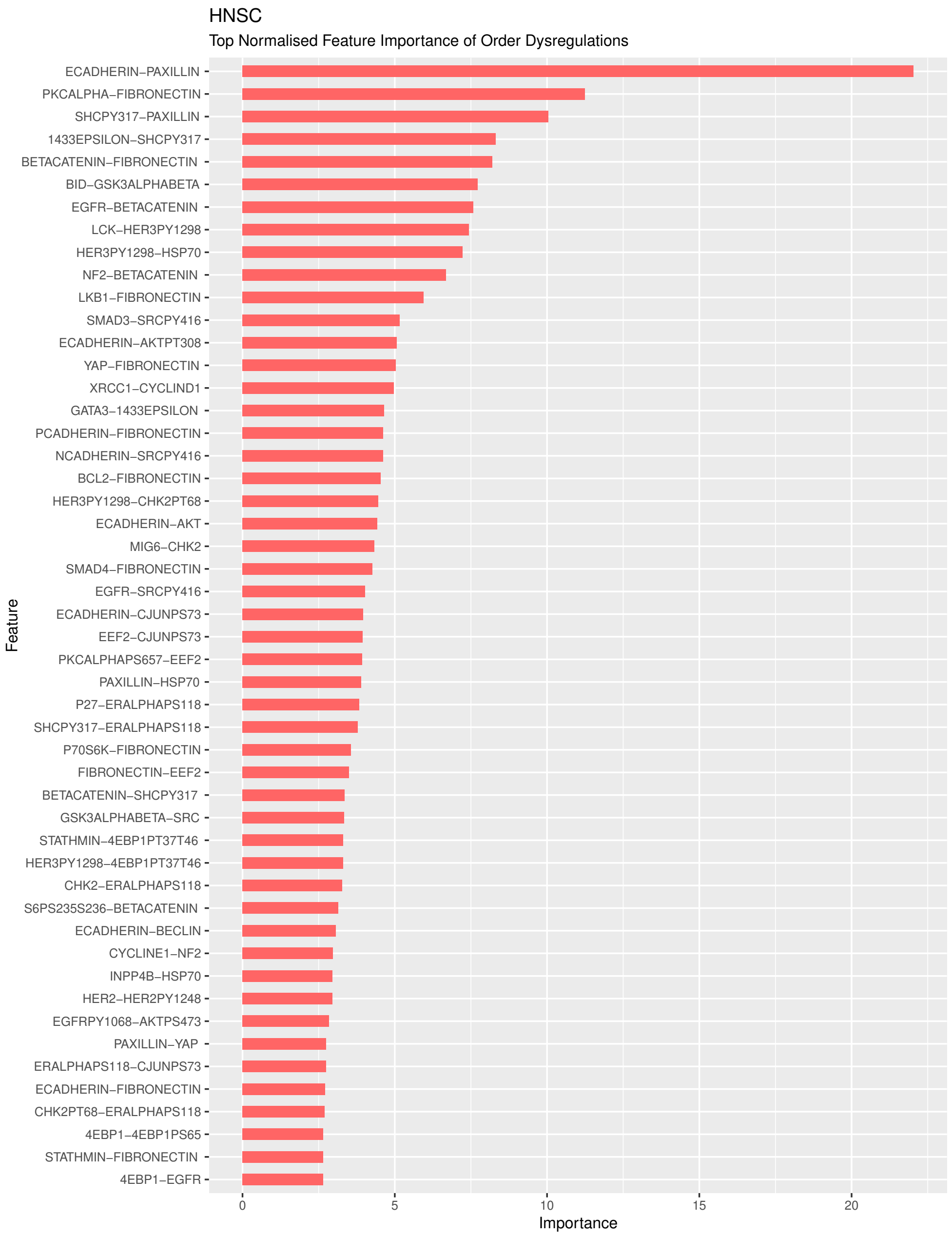

Figure P: Variable importance of significant pairwise ranking embeddings for head and neck squamous cell carcinoma.

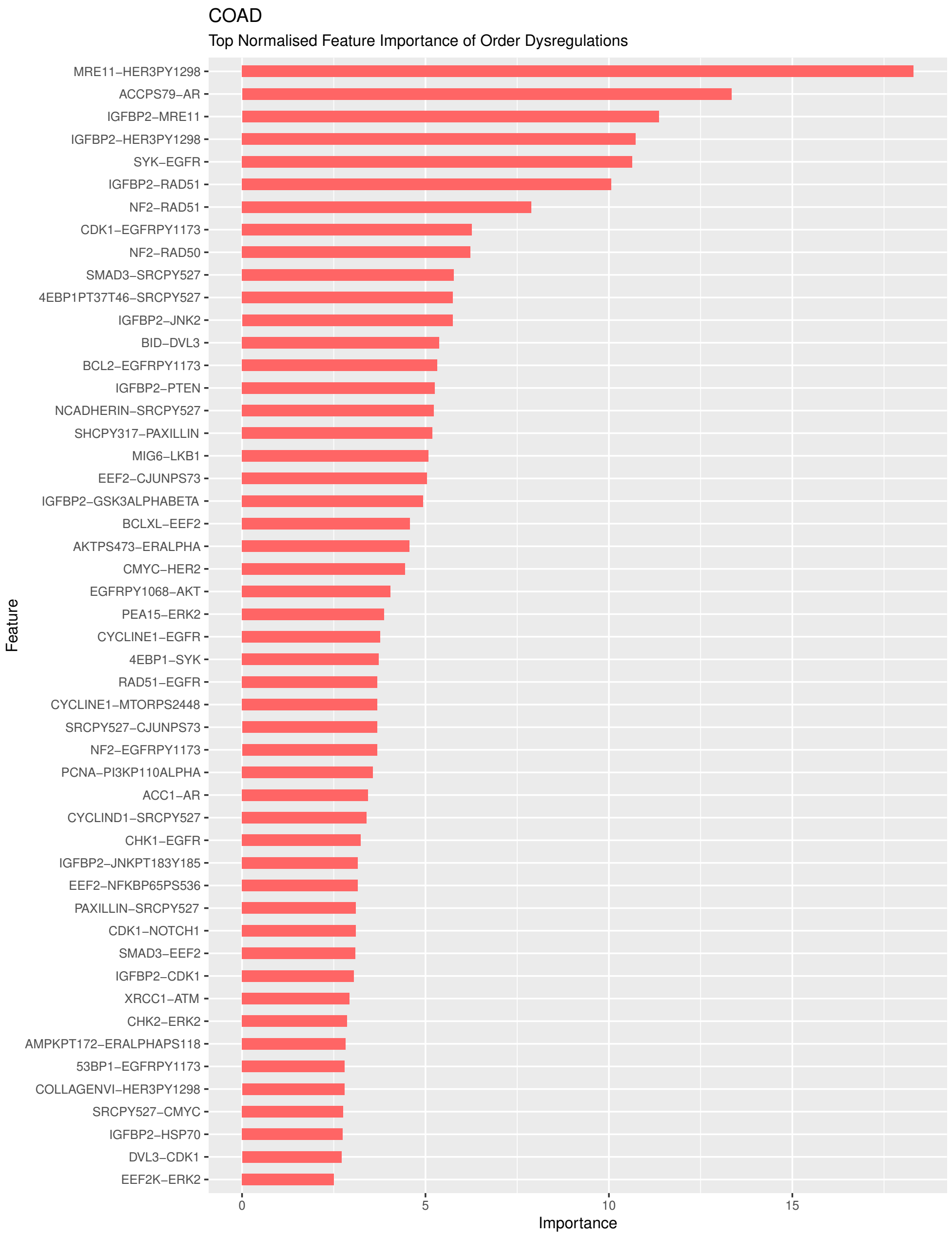

Figure Q: Variable importance of significant pairwise ranking embeddings for colon adenocarcinoma.

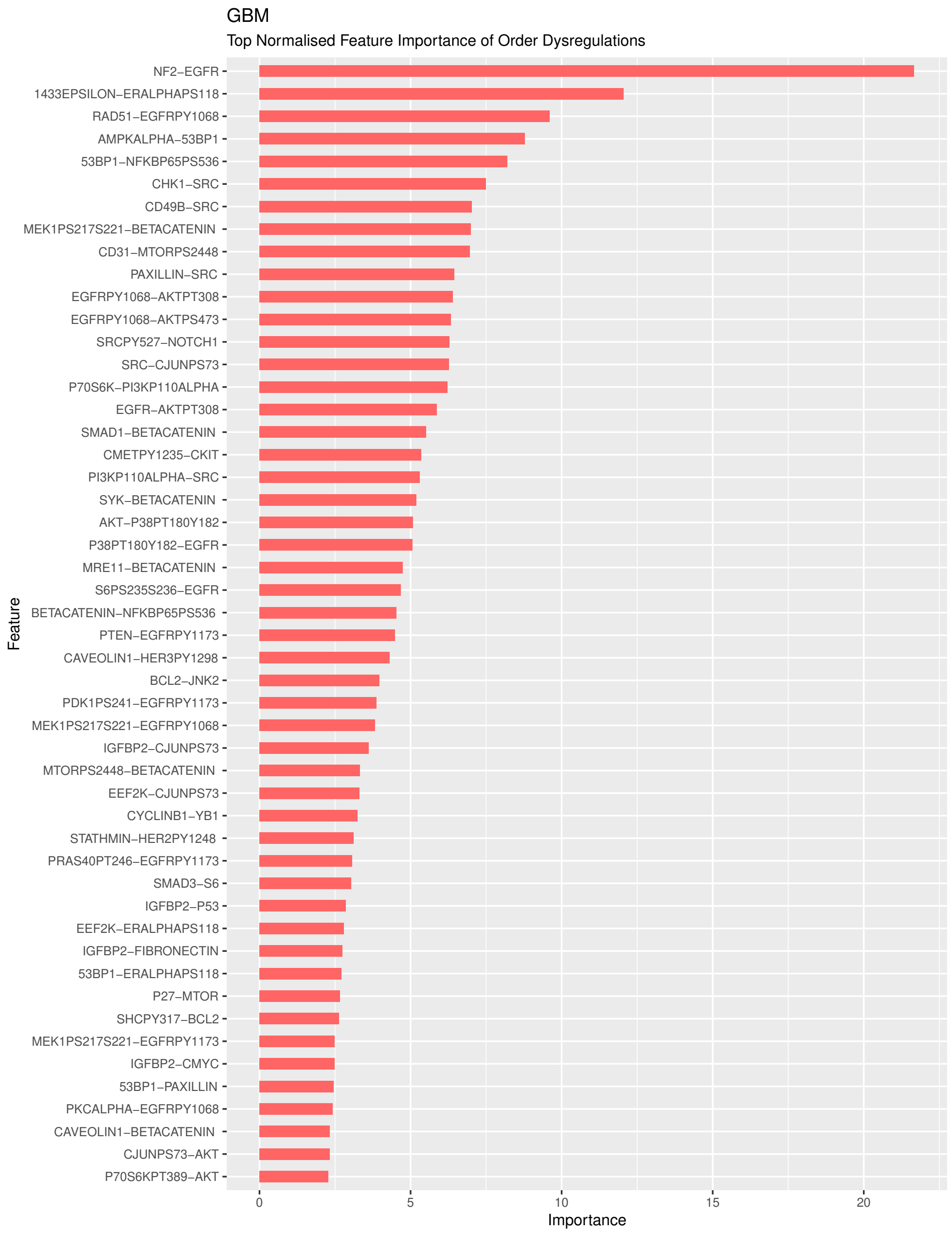

Figure R: Variable importance of significant pairwise ranking embeddings for glioblastoma multiforme.

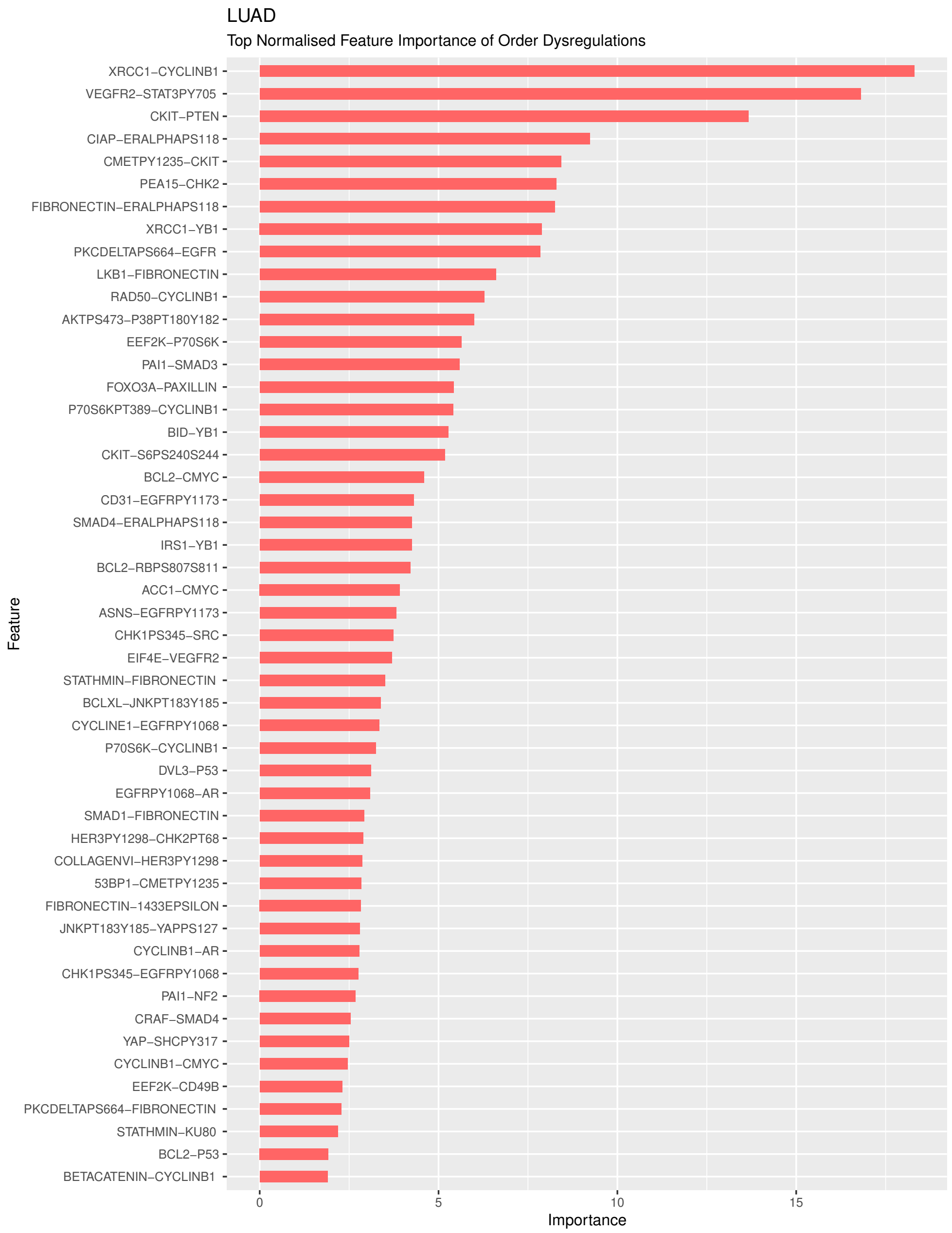

Figure S: Variable importance of significant pairwise ranking embeddings for lung adenocarcinoma.

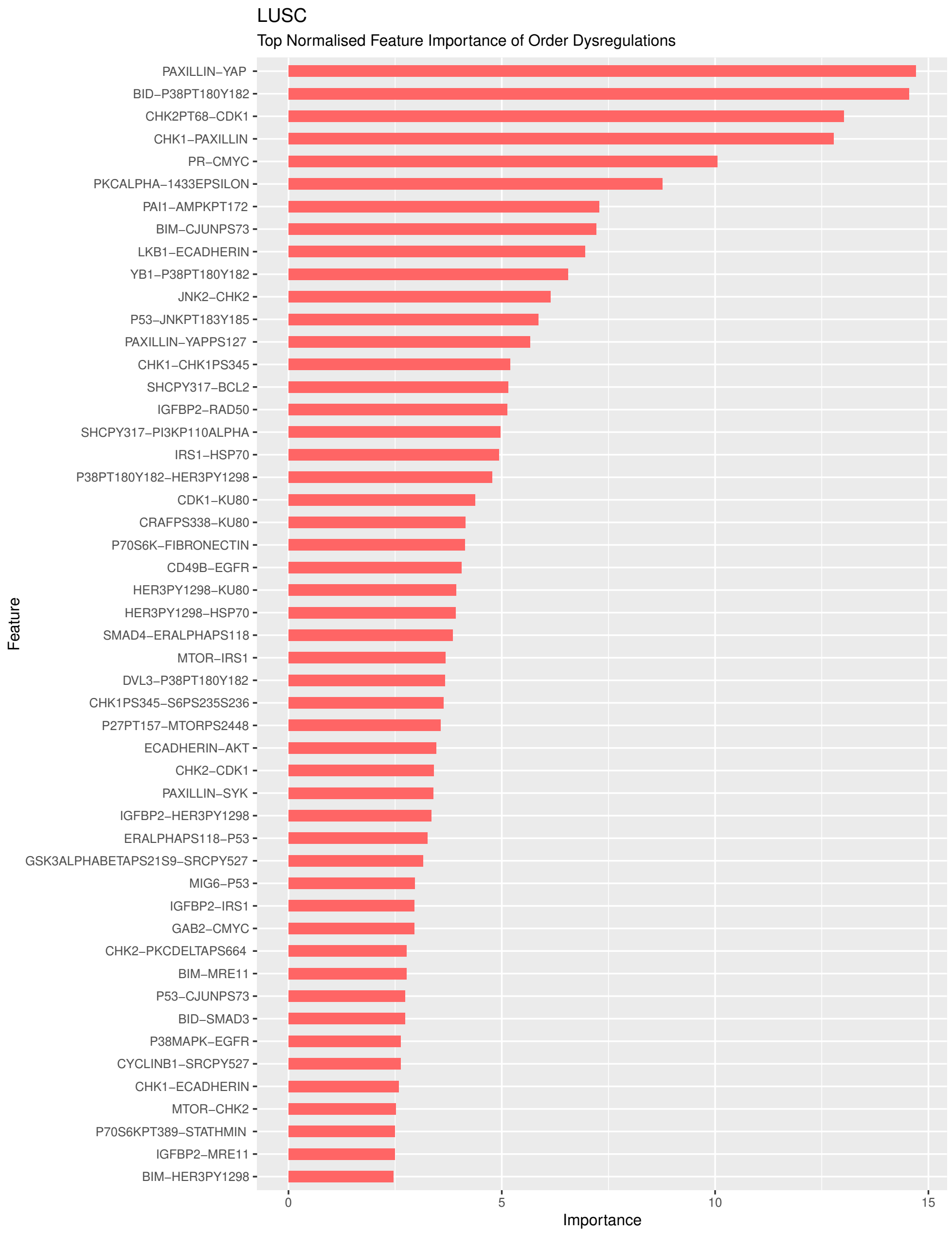

Figure T: Variable importance of significant pairwise ranking embeddings for lung squamous cell carcinoma.

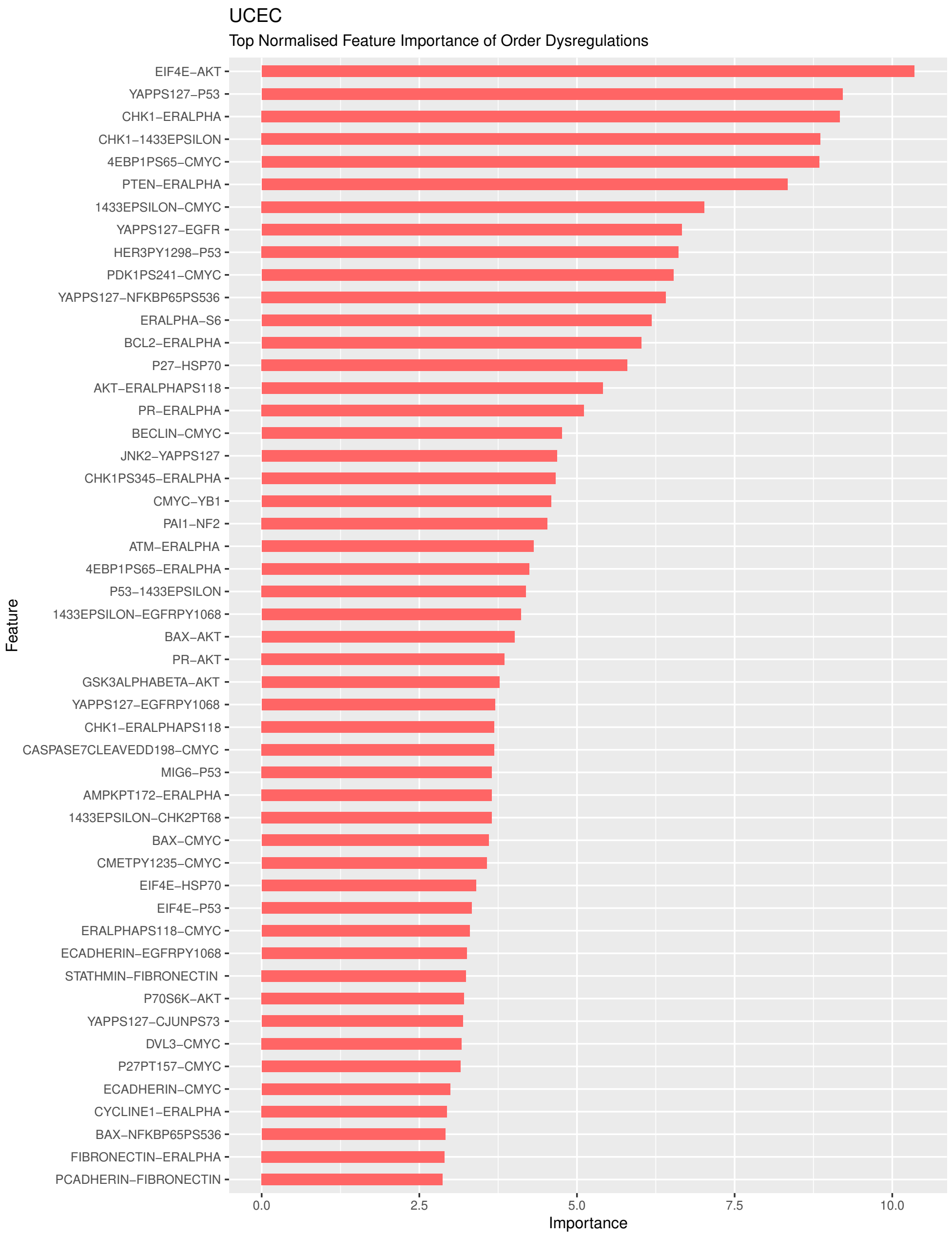

Figure U: Variable importance of significant pairwise ranking embeddings for uterine corpus endometrial carcinoma.

2.5 PRER Networks

KIRC

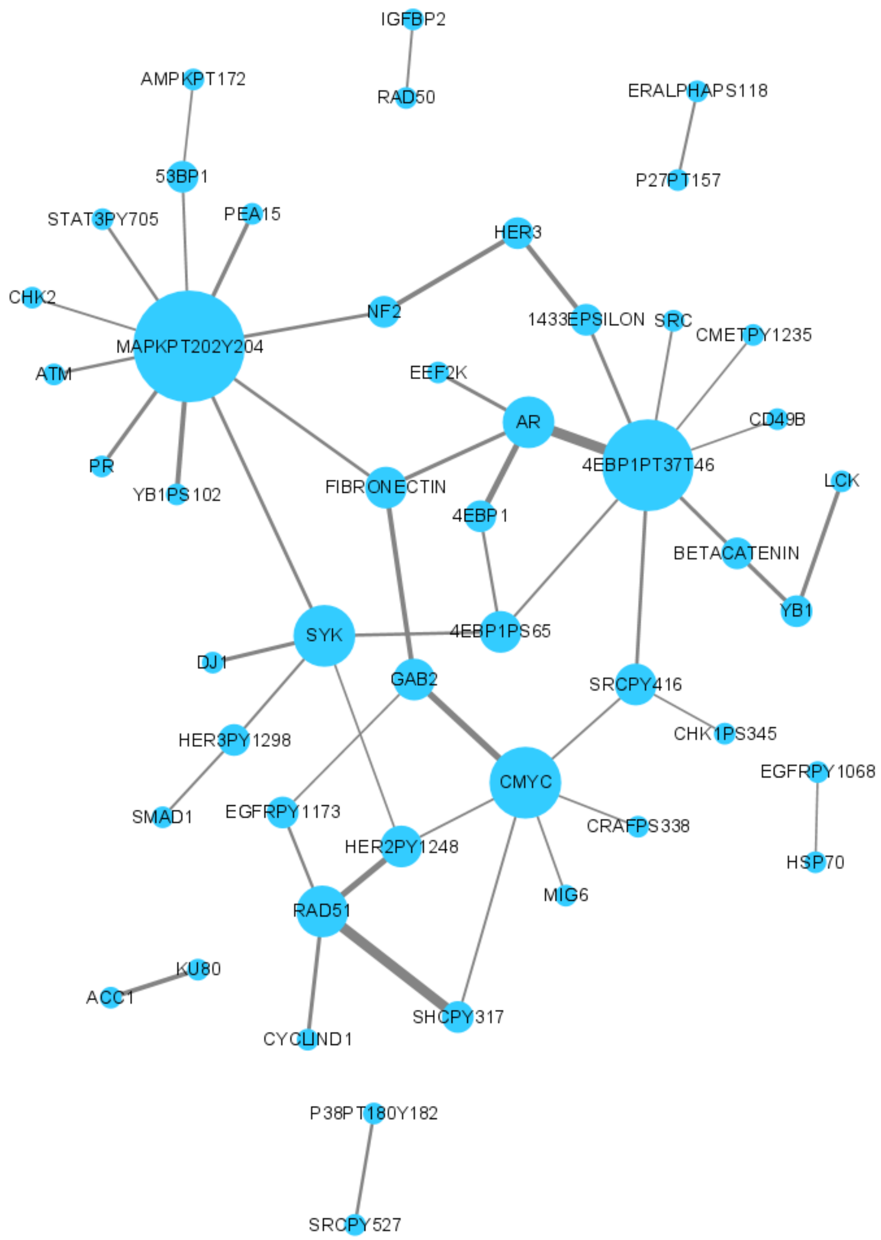

Figure V: Nodes represent proteins that appear in the top 50 pairwise ranking embeddings for kidney renal clear cell carcinoma; edges represent that two proteins participate in a pairwise rank order feature together.

BRCA

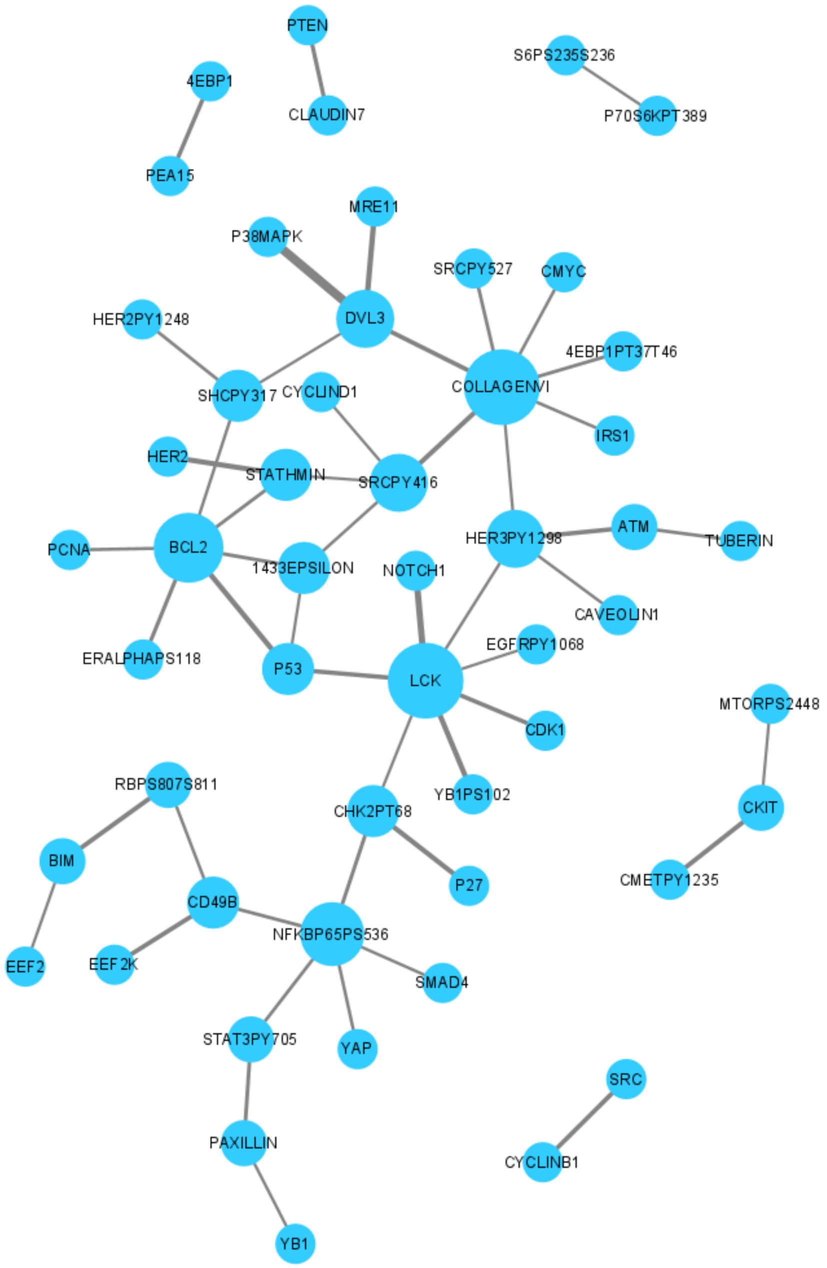

Figure W: Nodes represent proteins that appear in the top 50 pairwise ranking embeddings for breast invasive carcinoma; edges represent that two proteins participate in a pairwise rank order feature together.

BLCA

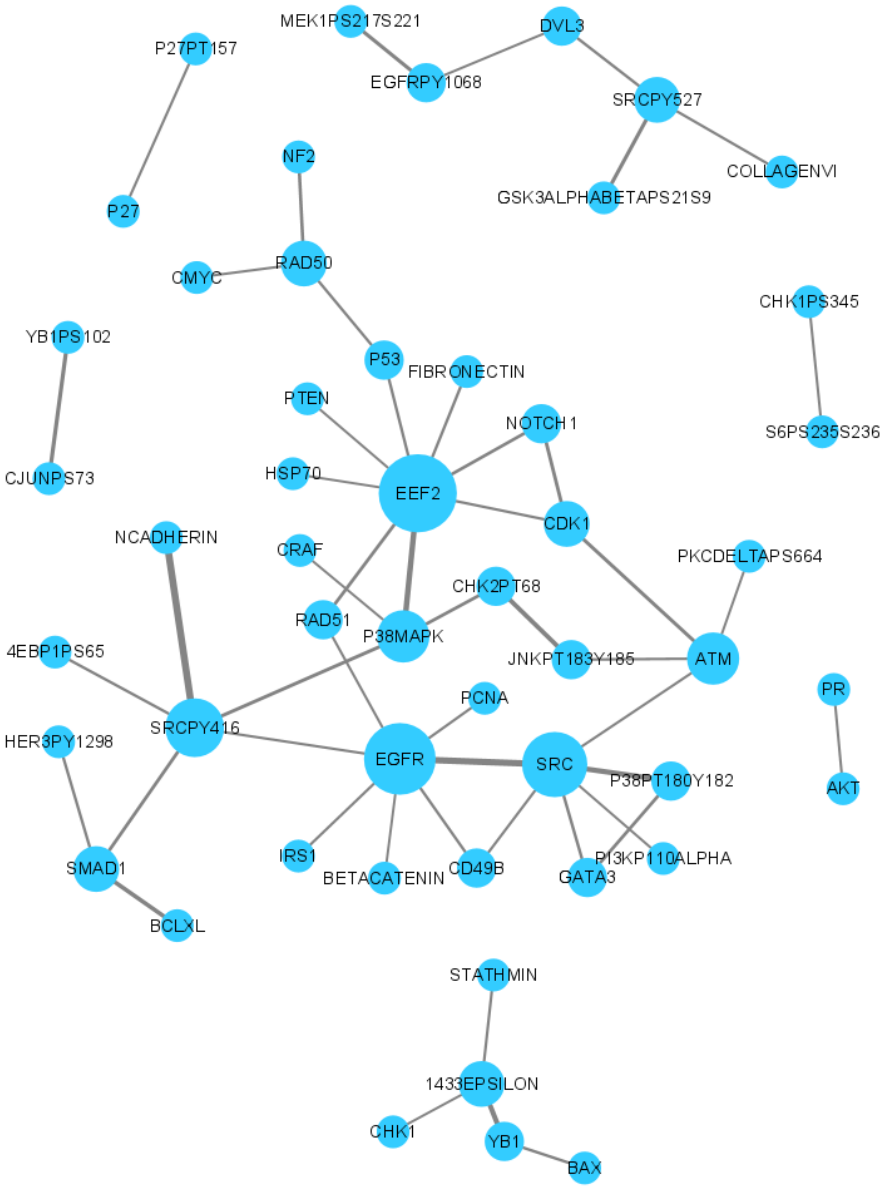

Figure X: Nodes represent proteins that appear in the top 50 pairwise ranking embeddings for bladder urothelial carcinoma; edges represent that two proteins participate in a pairwise rank order feature together.

HNSC

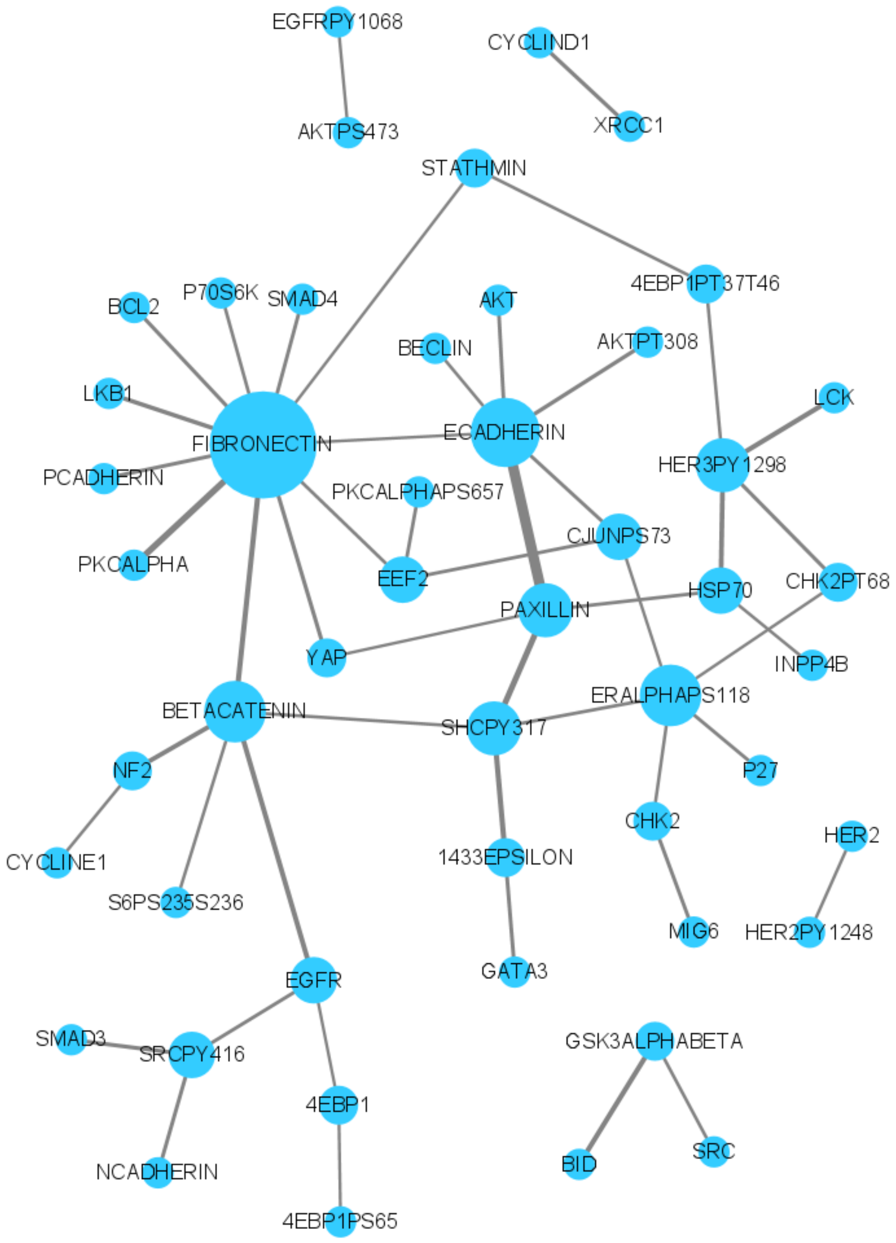

Figure Y: Nodes represent proteins that appear in the top 50 pairwise ranking embeddings for head and neck squamous cell carcinoma; edges represent that two proteins participate in a pairwise rank order feature together.

COAD

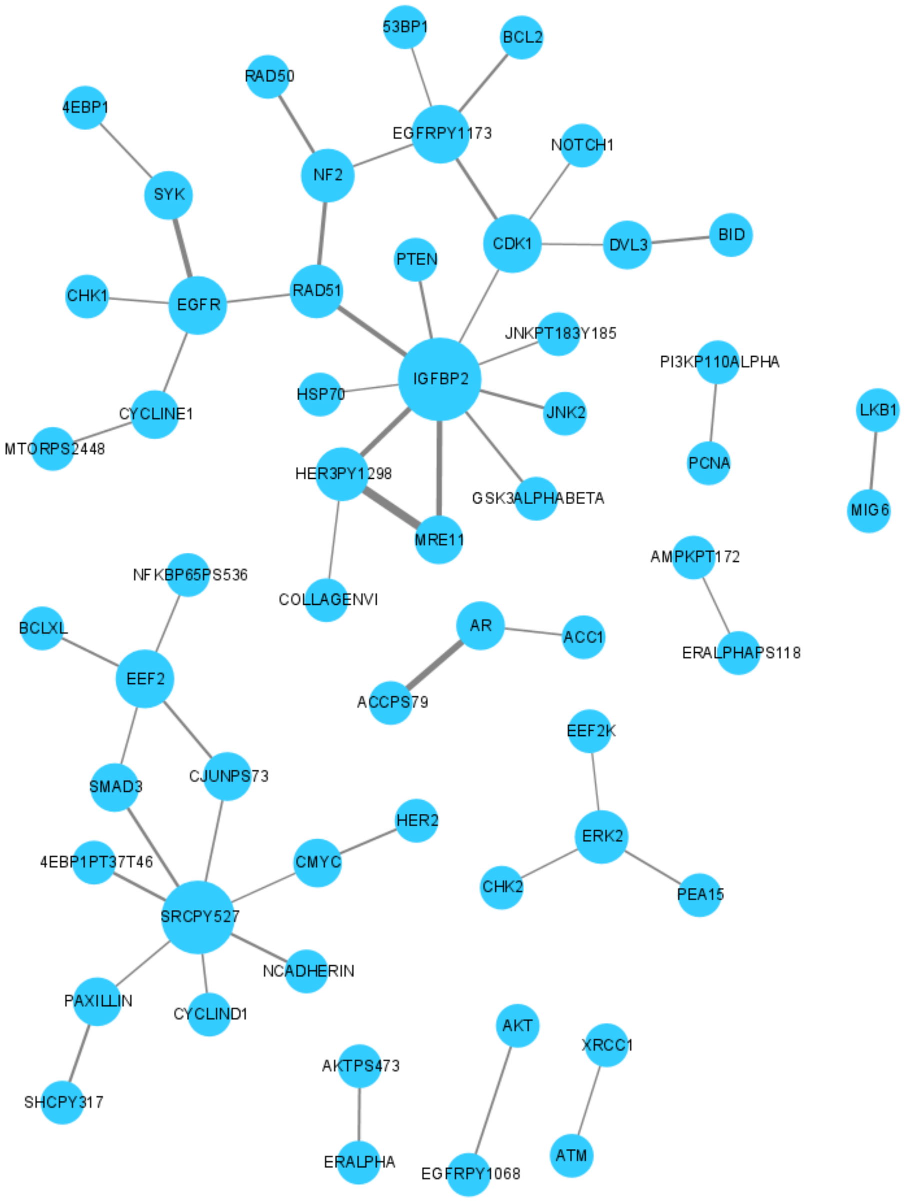

Figure Z: Nodes represent proteins that appear in the top 50 pairwise ranking embeddings for colon adenocarcinoma; edges represent that two proteins participate in a pairwise rank order feature together.

GBM

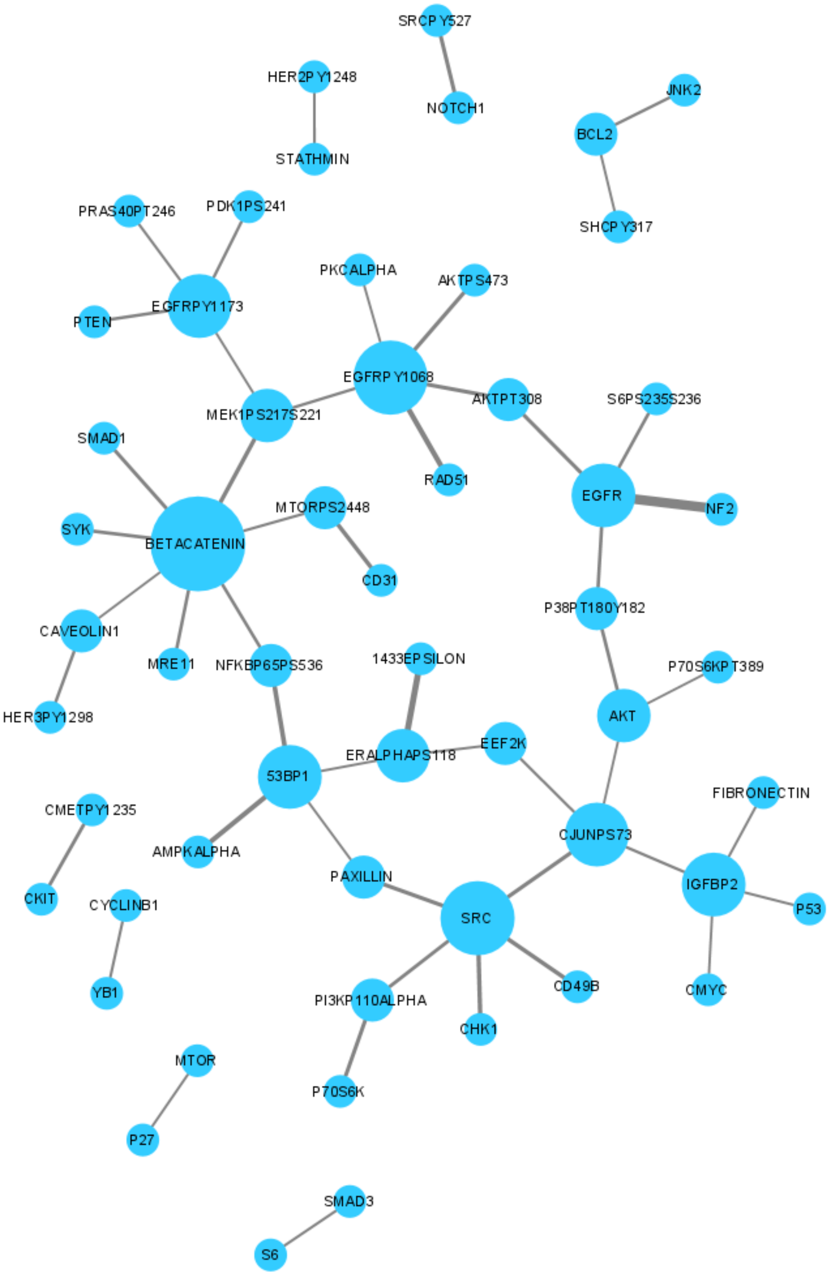

Figure AA: Nodes represent proteins that appear in the top 50 pairwise ranking embeddings for glioblastoma multiforme; edges represent that two proteins participate in a pairwise rank order feature together.

LUAD

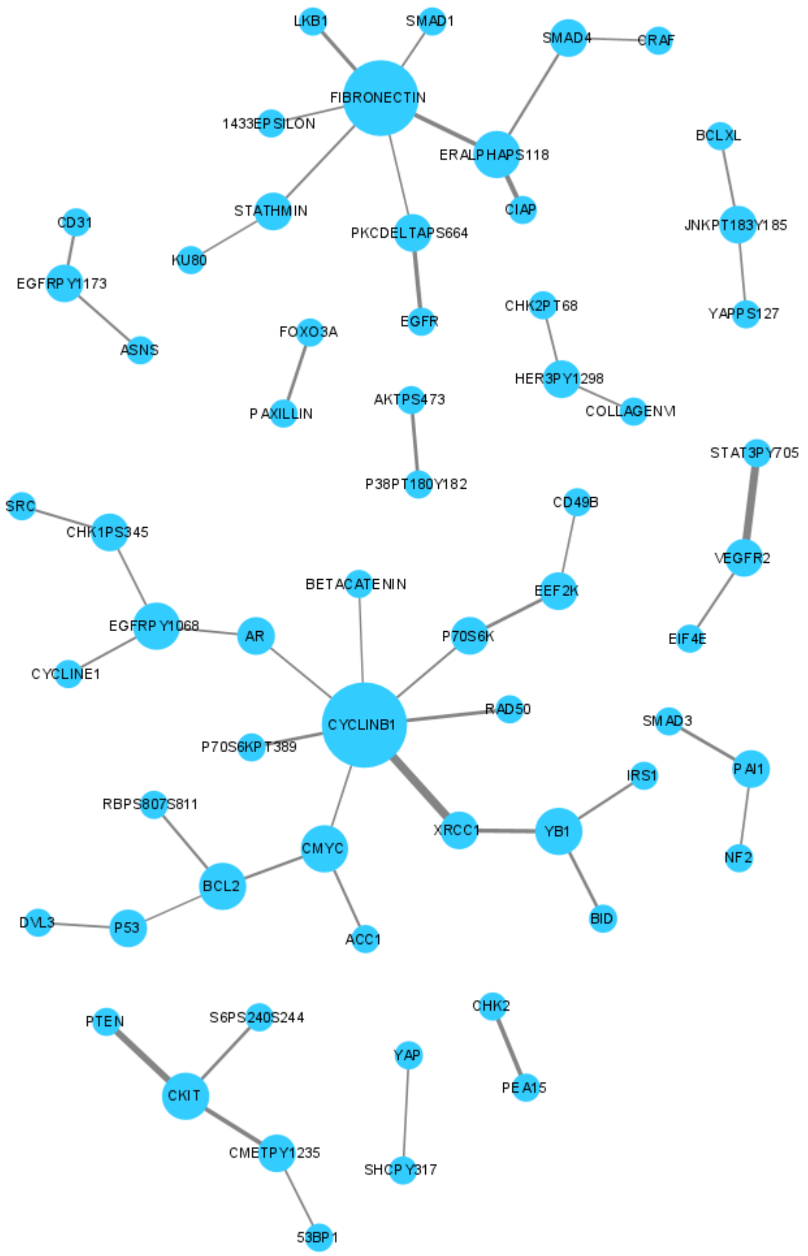

Figure AB: Nodes represent proteins that appear in the top 50 pairwise ranking embeddings for lung adenocarcinoma; edges represent that two proteins participate in a pairwise rank order feature together.

LUSC

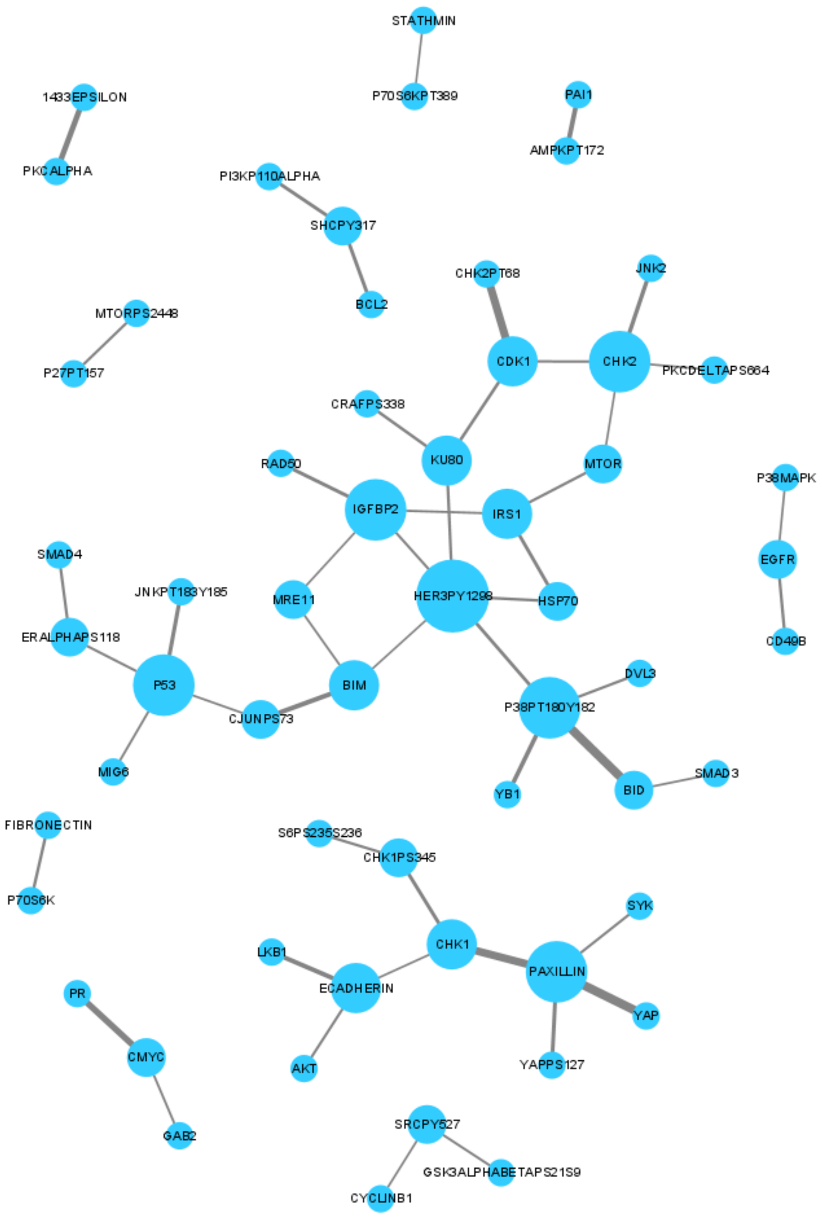

Figure AC: Nodes represent proteins that appear in the top 50 pairwise ranking embeddings for lung squamous cell carcinoma; edges represent that two proteins participate in a pairwise rank order feature together.

UCEC

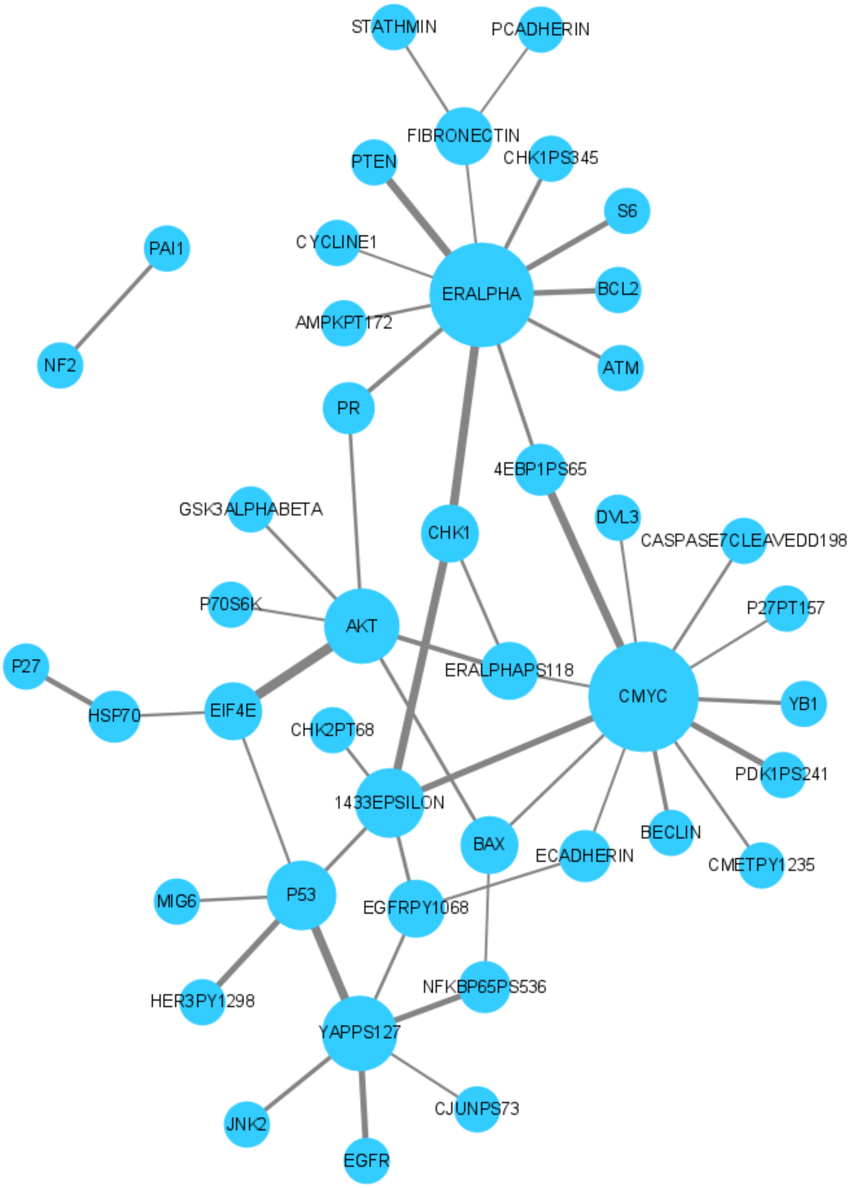

Figure AD: Nodes represent proteins that appear in the top 50 pairwise ranking embeddings for uterine corpus endometrial carcinoma; edges represent that two proteins participate in a pairwise rank order feature together.

2.6 Top Predictive PRER are Prognostic Biomarkers

To check the relevance of the PRER genes, we check whether the patients with different feature values are different in terms of their survival distribution. For a PRER protein pair, A-B, we group patients such that those patients with protein expression values A>B are in one group and the rest are in the other group. We then check if the survival distribution of these groups is different using the log-rank test. The Figure AEshows the age and sex adjusted KM plots for each cancer type and the top-ranked feature.

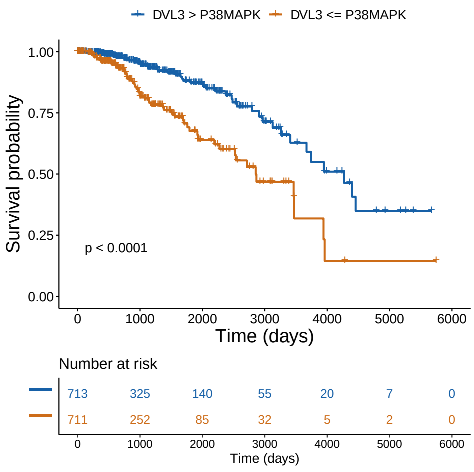

(a) BRCA

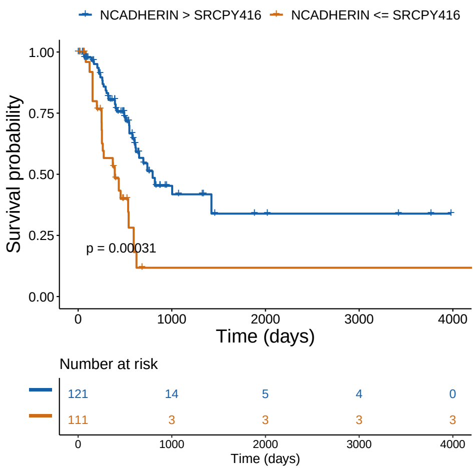

(b) BLCA

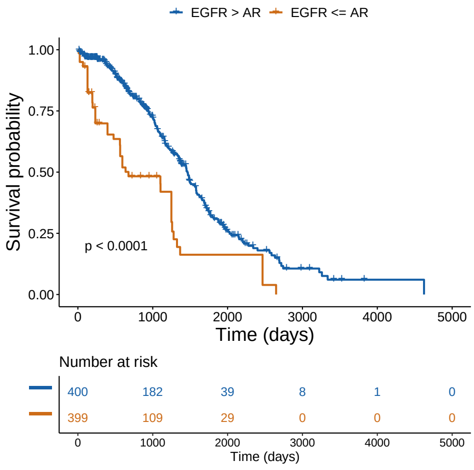

(c) OV

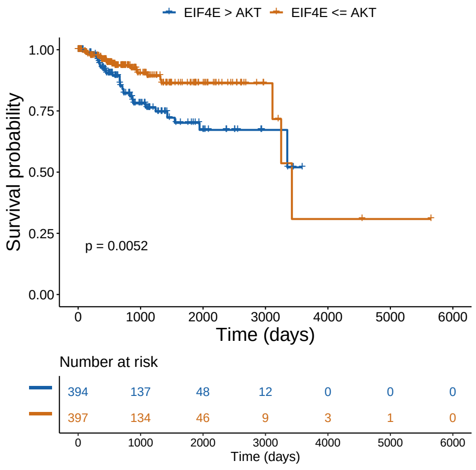

(d) UCEC

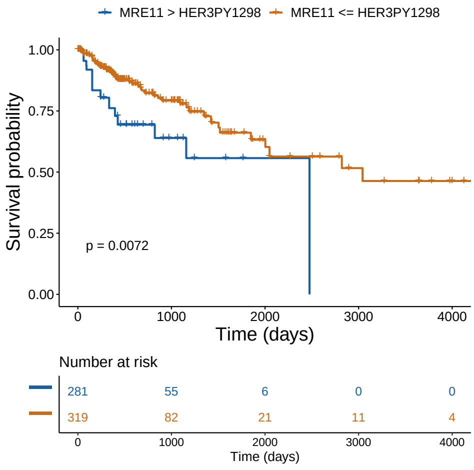

(e) COAD

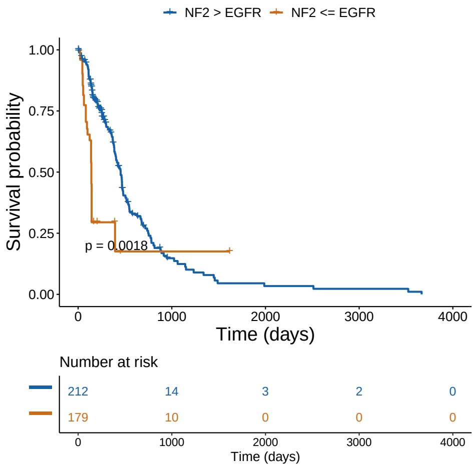

(f) GBM

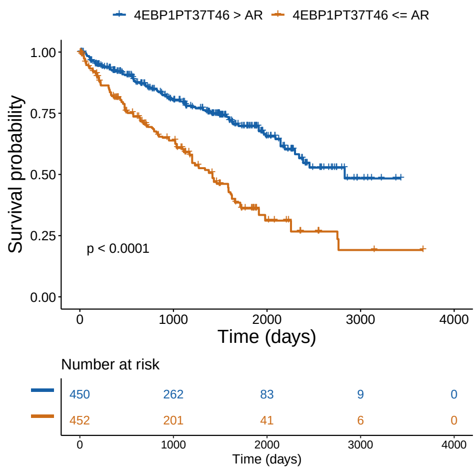

(g) KIRC

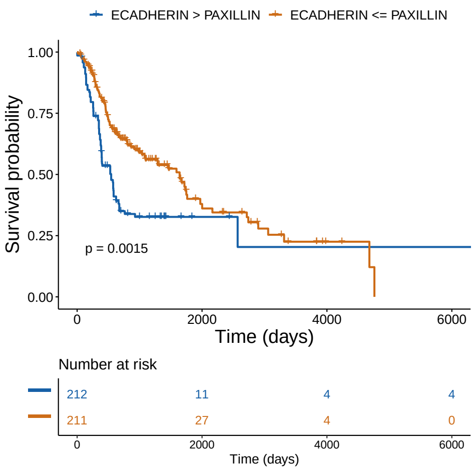

(h) HNSC

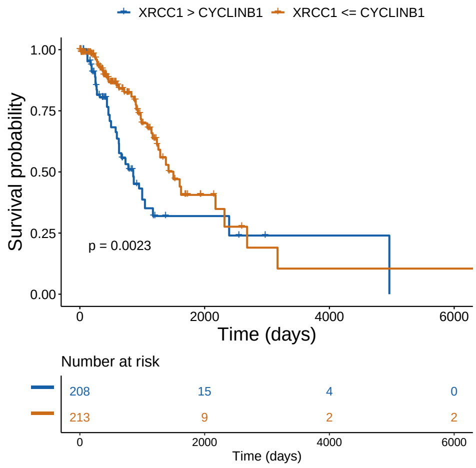

(i) LUAD

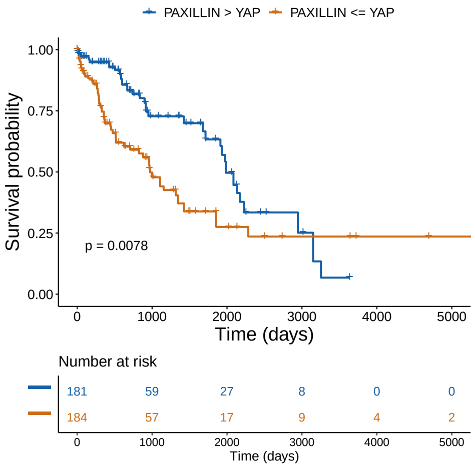

(j) LUSC

Figure AE: Age and sex adjusted Kaplan-Meier plot for each cancer type based on overall survival. Number at risk denotes the number of patients at risk at a given time, and p-value is calculated with the log-rank test.
